# ADAMTS9‐AS2 Disrupts Docetaxel‐Resistance in Castration‐Resistant Prostate Cancer via Stemness Suppression and Ferroptosis Induction

**DOI:** 10.1002/advs.202520838

**Published:** 2025-12-29

**Authors:** Ji Liu, Yan Gao, Yadong Guo, Junfeng Zhang, Wentao Zhang, Zhuoran Gu, Haotian Chen, Chengqi Jin, Peng Luo, Shiyu Mao, Yajuan Hao, Shuo Shi, Xudong Yao

**Affiliations:** ^1^ Department of Urology, School of Medicine, Shanghai Tenth People's Hospital Tongji University Shanghai P. R. China; ^2^ School of Medicine, Shanghai Tenth People's Hospital Tongji University Shanghai P. R. China; ^3^ School of Chemical Science and Engineering Department of Laboratory Medicine, Shanghai Tenth People's Hospital Tongji University Shanghai P. R. China; ^4^ The Department of Oncology, Zhujiang Hospital Southern Medical University Guangzhou Guangdong P. R. China

**Keywords:** prostate cancer, docetaxel resistance, ferroptosis, cancer stem cells, nanomaterial

## Abstract

Castration‐resistant prostate cancer (CRPC) chemotherapy resistance remained a significant clinical challenge. Prostate tumor stem cells (PCSCs) played a crucial role in chemotherapy resistance, but the underlying mechanisms were not fully understood. This study investigated how ADAMTS9‐AS2 reduced chemotherapy resistance in CRPC through a dual mechanism and explored the potential of polymeric materials targeting PCSCs and enhancing chemotherapy sensitivity. Key regulatory molecules of PCSCs were identified through mRNAsi‐based multi‐center patient cohorts. The effect of ADAMTS9‐AS2 on reducing docetaxel resistance in CRPC was assessed, and its mechanisms were further explored using in vitro and in vivo experiments. Finally, polymeric materials containing TGF‐β inhibitor, ferroptosis inducer, and miR‐182‐5p inhibitor were used to target PCSCs to improve chemotherapy sensitivity. ADAMTS9‐AS2 reduced CRPC chemotherapy resistance through dual mechanisms: (1) regulating FOXF2/TGF‐β2 axis to suppress PCSCs stemness; (2) encoding a short peptide that competitively retained more SLC7A11 in the cytoplasm than on the cytomembrane, thus promoting ferroptosis. Furthermore, polymeric materials targeting PCSCs significantly enhanced docetaxel sensitivity and inhibited tumor progression. ADAMTS9‐AS2 delayed docetaxel resistance by suppressing CRPC stemness and inducing ferroptosis. The use of polymeric materials targeting PCSCs offered a novel strategy to overcome CRPC chemotherapy resistance.

## Graphical abstract

This study uncovered a dual‐track mechanism by which ADAMTS9‐AS2 overcame chemoresistance in CRPC: suppressing cancer stemness through the FOXF2/TGF‐β2 pathway, and triggering ferroptosis via competitive SLC7A11 sequestration. Polymeric materials co‐targeting these vulnerabilities further boost docetaxel sensitivity, highlighting a compelling strategy to dismantle CRPC chemoresistance.

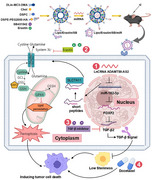

## 1 Introduction

Prostate cancer (PCa) had become one of the most common malignancies among men worldwide [1]. Androgen‐deprivation therapy (ADT) was the standard treatment for patients with advanced or metastatic PCa. However, after a period of ADT, most patients inevitably progressed to castration‐resistant prostate cancer (CRPC), a stage in which the disease rapidly advanced and was often accompanied by drug resistance, posing significant challenges for clinical management [2].

Since 2004, docetaxel (DTX)‐based chemotherapy had been recommended as the first‐line treatment for most symptomatic advanced PCa patients, significantly improving overall survival and quality of life [3]. Despite its initial effectiveness, the development of acquired resistance to DTX remained a major hurdle, leading to disease progression in many patients [4]. Thus, understanding the mechanisms underlying docetaxel resistance in CRPC was crucial for improving patient outcomes. Investigating these mechanisms would provide valuable insights into potential therapeutic strategies to overcome resistance and enhance the survival rate of CRPC patients.

Chemotherapy resistance was a complex process, and increasing evidence supported the crucial role of cancer stem cells (CSCs) in mediating chemotherapy resistance [5, 6]. CSCs were a subpopulation of cells within the tumor that possessed stem cell‐like properties, including self‐renewal and the ability to differentiate into multiple cell types [7]. Studies had confirmed the presence of prostate cancer stem cells (PCSCs) in CRPC tissue, suggesting their involvement in disease progression and therapeutic resistance [8]. These PCSCs were typically resistant to chemotherapy, allowing them to survive and contribute to tumor relapse and progression [9]. Studies, including research from our own team, had indicated that targeting CSCs might be a promising approach to inhibit the progression of CRPC [10, 11, 12, 13]. Therefore, elucidating the mechanisms of chemotherapy resistance from the perspective of cancer stem cells held significant research value for improving the treatment of CRPC.

Due to the relatively low abundance of CSCs, studying them in depth remained challenging. However, the single‐class logistic regression (OCLR), a machine learning algorithm, had been proven to be an effective method for quantifying the stemness index of tumors. This method primarily relied on two independent metrics for quantification [14]. A study published in *Cell* demonstrated that the stemness index (mRNAsi), based on transcriptomics, could measure the dedifferentiation (stemness) level of a sample [15]. In contrast, the DNA‐based stemness index (mDNAsi), derived from epigenetic features, reflected the epigenetic characteristics of the sample [16]. Previous studies had shown that OCLR was highly effective in various malignancies, making its application to assess prostate cancer stem cells particularly meaningful.

In recent years, with advancements in genomics technologies, there had been significant breakthroughs in the functional research of long non‐coding RNAs (LncRNAs). Increasing evidence suggested that LncRNAs not only played a role in transcriptional regulation but also had potential protein‐coding functions—capable of translating into small functional micropeptides. These micropeptides encoded by LncRNAs exhibited various important functions in cancer biology, such as regulating cell proliferation, inhibiting apoptosis, and promoting tumor invasion and metastasis [17]. For instance, some micropeptides encoded by LncRNAs had been found to directly participate in the regulation of cancer‐related signaling pathways or influence tumor progression through interactions with other proteins [18].ADAMTS9‐AS2 was an antisense transcript of the tumor‐suppressor gene ADAMTS9, with both located on human chromosome 3p14.1 and forming a complementary pair. It had been reported to exert inhibitory effects on tumor initiation, progression, and metastasis in esophageal cancer [19], lung adenocarcinoma [20], and oral squamous cell carcinoma [21].

Ferroptosis was a form of iron‐dependent cell death characterized by the accumulation of lipid peroxides, mediated by reactive oxygen species (ROS), which ultimately led to cell death [22]. SLC7A11 was an important component of the system Xc‐, playing a critical role in suppressing ferroptosis by regulating the synthesis of glutathione (GSH) within cells [23]. Overexpression of SLC7A11 in many cancer types had been associated with tumor cell resistance to ferroptosis, making it a key mechanism for tumor cell survival and chemotherapy resistance [24].

Polymeric materials had shown significant potential in overcoming cancer chemotherapy resistance. These materials could be designed with multifunctional properties, enabling precise drug delivery, controlled release, and multi‐target synergistic effects, thus significantly enhancing the anticancer efficacy. For example, studies had demonstrated that integrating chemotherapy drugs, gene therapy molecules (such as siRNA or peptides), and targeting ligands into a single polymeric nanoparticle could effectively overcome tumor resistance and reduce drug side effects [25]. Additionally, polymeric materials could carry drug combinations or combine with ferroptosis inducers to induce programmed cell death in tumor cells, thereby reversing drug resistance [26]. These nanomaterials could also be further modified with ligands targeting cancer stem cells, ensuring that the drugs specifically target drug‐resistant cancer cell populations [27]. Therefore, the multifunctional design of polymeric materials provided a promising new strategy for overcoming chemotherapy resistance in cancer and demonstrated great potential for clinical applications.

In CRPC, the interrelationships among DTX resistance, PCSCs, and the lncRNA ADAMTS9‐AS2 remain poorly understood. In this study, we observed elevated expression of stem cell markers in DTX‐resistant CRPC samples, suggesting a potential link between cancer stemness and chemoresistance. Through weighted gene co‐expression network analysis (WGCNA) based on mRNAsi, we identified lncRNA ADAMTS9‐AS2 as a key regulator, which was significantly downregulated in PCSCs and PCa tissues. Functional characterization revealed that lncRNA ADAMTS9‐AS2 operated via a dual mechanism: on one hand, it encoded a short peptide that interacted with SLC7A11 to promote ferroptosis; on the other hand, it mediated the regulation of the TGF‐β pathway by FOXF2, thereby suppressing PCSC stemness and reducing docetaxel resistance. Building on these findings, we developed a novel polymeric nanomaterial specifically designed to target prostate cancer stem cells. This therapeutic platform incorporated FOXF2‐promoting microRNA inhibitors, TGF‐β pathway inhibitors, and ferroptosis inducers, demonstrating potent efficacy in overcoming DTX resistance and inhibiting malignant progression in CRPC models. Our results provided a promising translational strategy for addressing docetaxel resistance in CRPC patients.

## 2 Materials and Methods

### 2.1 Clinical Samples

A total of 10 pairs of hormone‐sensitive PCa tissues and CRPC tissues were collected from Shanghai Tenth People's Hospital, Tongji University (Shanghai, China), with confirmation of all tissue samples by a pathologist. The study was conducted in accordance with the Declaration of Helsinki, and ethical approval was obtained from the Ethical Committee of Shanghai Tenth People's Hospital (Approval number: SHSY‐IEC‐4.1/20‐22/01), with written informed consent obtained from all patients. The collected tissues were immediately snap‐frozen in liquid nitrogen after resection for subsequent RNA extraction. Additionally, transcriptomic data and clinical information for 499 prostate cancer cases, 35 normal tissue samples, and 92 metastatic castration‐resistant prostate cancer (mCRPC) samples were retrieved from The Cancer Genome Atlas (TCGA) database. Detailed information about the clinical samples was provided in Table S1.

### 2.2 mRNAsi Calculation and Data Processing

The mRNAsi was an index used to assess the stemness characteristics of tumor samples based on transcriptomic data, reflecting the stem cell‐like properties of the samples. To calculate mRNAsi, we adopted the method described by Malta et al. [15], which analyzed gene expression profiles of PCa samples from the TCGA database and compared them with known stem cell gene sets. Machine learning techniques such as LASSO regression were used to select the most representative stemness‐related genes. The resulting mRNAsi value reflected the stemness level of each sample. In this study, mRNAsi data from TCGA prostate cancer samples were merged with clinical data using a Strawberry Perl script, and cases that did not match with normal tissues were removed. All data processing and analysis were conducted using standard bioinformatics tools and scripts to ensure accuracy and consistency.

### 2.3 Weighted Gene Co‐expression Network Analysis

Using the “WGCNA” R package v1.69, gene co‐expression networks were constructed based on the mRNA expression matrix. First, a Pearson correlation coefficient matrix was generated to assess the pairwise similarity of gene expression. The appropriate β value was determined at a degree of independence (R^2^) of 0.9, ensuring the creation of a scale‐free network. Next, the weighted adjacency matrix was transformed into a topological overlap matrix (TOM), which quantified network connectivity. A gene dendrogram was constructed with a minimum module size of 50, and highly similar modules with a correlation greater than 0.7 were merged. Genes with similar expression patterns were then grouped into gene modules using a mean linkage hierarchical clustering method based on TOM measurements. Gene modules were represented by their module eigengene (ME) expression. The module most strongly associated with mRNAsi (|r| = 0.82) was selected for further analysis. Gene Ontology (GO) and Kyoto Encyclopedia of Genes and Genomes (KEGG) analyses were performed to identify the potential molecular pathways and biological processes related to the mRNAsi‐associated genes.

### 2.4 Cell Culture

PCa cell lines used in this study, including PC3 (CRL‐1435), DU145 (HTB‐81), and the normal human prostate cell line RWPE‐1 (CRL‐3607), were obtained from the Chinese Academy of Sciences (Shanghai, China). Cells were cultured in RPMI‐1640 medium, supplemented with 10% fetal bovine serum (FBS) (Gibco, New York, USA) and penicillin/streptomycin (100 µg/mL) (HyClone, Logan, USA). The RWPE‐1 cells were cultured in Defined Keratinocyte SFM (Invitrogen, Waltham, USA). All cells were maintained at 37°C in a 5% CO_2_ incubator. The details of siRNA, shRNA, miRNA mimics or inhibitors, and recombinant plasmids of lncRNA ORFs used in the study were provided in Table S3.

### 2.5 Enrichment and Sphere‐Forming Assay of CSCs

PC3 and DU‐145 cells were cultured in serum‐free Dulbecco's Modified Eagle Medium/F12 (DMEM/F12, Gibco, New York, USA), supplemented with insulin (Sigma‐Aldrich, City of Saint Louis, USA), 20 ng/mL epidermal growth factor (EGF, Peprotech, Suzhou, China), 10 ng/mL basic fibroblast growth factor (bFGF, Peprotech, Suzhou, China), and 0.4% bovine serum albumin (BSA, Yeason, Shanghai, China). After 2 weeks of culture, 3 × 10⁵ cells were seeded into six‐well plates for CSC enrichment, with medium replacement every other day. Morphological changes of the CSCs were observed under a microscope. For the sphere‐forming assay, 1000 cells were seeded into ultra‐low attachment six‐well plates and cultured in the same medium. After 2 weeks, the spheres were counted and photographed. Cell clusters larger than 50 µm in diameter were considered as CSCs.

### 2.6 Co‐Immunoprecipitation (Co‐IP) Assay

Cells were lysed on ice for 30 min with lysis buffer (Epizyme, Shanghai, China). Meanwhile, 50 µL of Dynabeads Protein G (Yeasen, China) was incubated with 5 µg of antibody at room temperature for 1 h. The protein lysate with the antibody‐beads complex was incubated overnight at 4°C with gentle shaking. After three washes with lysis buffer, bound proteins and 10% of the input were analyzed by Western blot. Silver staining was then performed, and gel strips were sent to Shanghai OE Biotech Co., Ltd (Shanghai, China) for mass spectrometry analysis.

### 2.7 3D Matrigel Drop Invasion Assay

1 × 10⁵ cancer cells expressing GFP were suspended in 10 µL of 100% Matrigel and seeded as droplets in 24‐well plates. Fluorescence images were captured on day 0 and day 6 using a fluorescence microscope. The culture medium was refreshed every three days. Cell invasion and migration from the Matrigel droplet were quantified based on RFP signals using ImageJ software. GFP fluorescence was represented by an orange pseudocolor. All experiments were conducted in triplicate.

### 2.8 Lipid Reactive Oxygen Species (ROS) Assay

To detect lipid ROS in PCa cells, the lipid peroxidation sensor C11‐BODIPY 581/591 (RM02821, Abclone, China) was employed. Cells were treated with various agents for 96 h, followed by a 30‐min incubation with C11‐BODIPY probe at 37°C in the dark. Afterward, the cells were washed three times with PBS, and flow cytometry was performed to measure the fluorescence of C11‐BODIPY (FITC, 484 nm/510 nm).

### 2.9 Ferrous Ion Fluorescence Assay

Cells were seeded in Petri dishes and cultured according to the instructions of the Ion Fluorescent Probe‐Mito‐FerroGreen kit (Tongren Co., Ltd, Shanghai, China), and incubated in a 5% CO_2_ atmosphere at 37°C. After removing the culture medium, the cells were washed three times with HBSS buffer or serum‐free medium. They were then incubated at 37°C with 5% CO_2_ for 30 min. Following this, the supernatant was removed, and the cells were washed three times again with HBSS buffer or serum‐free medium. The cells were subsequently incubated with activators (such as Erastin or RSL3) in fresh medium at 37°C with 5% CO_2_. The fluorescence images were captured using confocal microscopy.

### 2.10 Liposome (LPs) Synthesis

Cationic lipid DLin‐MC3‐DMA (MC3), DSPC, cholesterol (Chol), DSPE‐PEG2000‐HA, and lipophilic drugs (Erastin or TGF‐β inhibitor) were dissolved in a mixed solution of methanol and chloroform (v/v = 1:3). The solution was then transferred to a round‐bottom flask and subjected to rotary evaporation, allowing the solution to form a thin film on the inner wall of the flask. Deionized water was added to the flask containing the film, and the mixture was continuously stirred to redissolve the film. After sonication, liposomes were formed. For liposomes loaded with miRNA, miRNA inhibitors were dissolved in DEPC water and vortexed into the liposomes. Detailed information on the liposome formulation was presented in Table 1.

**TABLE 1:** Details of LPs formulations, including determinate molar ratio of each component, the weight ratio of MC3 to Erastin, TGF‐β inhibitor and miRNA‐inhibitor.

Name	Molar Ratios				MC3/Erastin (wt/wt)	MC3/TGF‐β inhibitor (wt/wt)	MC3/miRNA‐inhibitor (wt/wt)
	MC3	DSPC	Chol	DSPE‐PEG2000‐HA			
1	50	10	39	0.5	−	−	−
2	50	10	39	0.5	10	−	−
3	50	10	39	0.5	−	10	−
4	50	10	39	0.5	−	−	159
5	50	10	39	0.5	10	10	159

### 2.11 Drug Loading Efficiency of Liposomes

High‐performance liquid chromatography (HPLC) was employed to determine the drug loading of Erastin and TGF‐β inhibitor. Encapsulation efficiency (EE%) was calculated as:

(1) $$\mathrm{EE}\left(\%\right)=\frac{\mathrm{Amount}\;\mathrm{of}\;\mathrm{drug}\;\mathrm{encapsulated}}{\mathrm{Total}\;\mathrm{of}\;\mathrm{drug}\;\mathrm{encapsulated}}\times 100\%$$

Here, the “Amount of drug encapsulated” referred to the quantity of drug that was actually incorporated into the LPs, and the “Total amount of drug encasulated” was the initial amount of drug used in the preparation of the LPs.

The drug loading efficiency (DLE) was calculated as:

(2) $$\mathrm{DLE}\left(\%\right)=\frac{W_{\mathrm{encapsulated}}}{W_{\mathrm{total}}}\times 100\%$$

Here, *W*_encapsulated_ was the weight of the drug encapsulated within the LPs, *W*_total_ was the total weight of the LPs formulation.

### 2.12 Investigation of Liposomal Drug Release

To elucidate the drug release characteristics of liposomes under different physiological and pathological conditions, buffer solutions with pH values of 7.4 and 5.6 were utilized to simulate physiological conditions and the tumor microenvironment, respectively. Liposome suspensions were prepared in the respective buffer solutions (pH 7.4 and 5.6) at a predetermined concentration. The liposome suspensions were incubated at 37°C in a shaking water bath to mimic in vivo conditions. At predetermined time intervals (e.g., 0.5, 1, 2, 4, 8, 12, and 24 h), aliquots of the release medium were withdrawn and replaced with an equal volume of fresh buffer solution to maintain a constant volume and sink conditions. The withdrawn samples were filtered through a 0.22 µm syringe filter to remove any residual liposome particles. The filtrates were then analyzed using HPLC to quantify the drug content released from the liposomes. The drug release rate was calculated by comparing the drug concentration in the release medium at each time point with the initial drug loading in the liposomes.

### 2.13 Animal Experiments

All animal experiments were performed in compliance with the National Regulations of China for the Care and Use of Laboratory Animals. The protocols were approved by the Ethics Committee of Shanghai Tenth People's Hospital, Tongji University (Approval No. SHDSYY‐2021‐3028). Male C57BL/6 and nude mice, purchased from Vital River Laboratory Animal Technology Co., Ltd. (Beijing, China), were used in the experiments. For the subcutaneous tumor formation assay, 2 × 10⁷ ADAMTS9‐AS2‐overexpressing RM‐1‐Lucifer cells were suspended in DMEM and Matrigel (1:1 v/v) and injected subcutaneously into the left flank of C57BL/6 mice. When tumor volumes reached approximately 100 mm^3^, mice in the experimental group received 3.22 × 10^−^^3^ g/kg docetaxel via intraperitoneal injection, while the control group was treated with an equivalent volume of DMSO. The treatment was repeated seven times, and tumor growth was monitored using in vivo fluorescence small animal imaging. For the lung metastasis assay, 1 × 10⁶ ADAMTS9‐AS2‐overexpressing, docetaxel‐resistant DU145‐Lucifer cells were injected into the tail vein of BALB/c nude mice, followed by 1.616 × 10^−^^3^ g/kg docetaxel treatment via intraperitoneal injection every two days. After two weeks, lung metastasis was assessed using in vivo fluorescence small animal imaging. For the materials used in the subcutaneous tumor formation assay, 2 × 10⁷ docetaxel‐resistant RM‐1 cells were suspended in DMEM and Matrigel (1:1 v/v), and injected subcutaneously into the left flank of both nude and C57BL/6 mice. When tumor volumes reached approximately 150 mm^3^, mice were randomly divided into five groups (n = 5 per group) and received 3.22 × 10^−^^3^ g/kg docetaxel via intraperitoneal injection. The groups included: (1) control: 100 µL Lipo via retro‐orbital injection; (2) 100 µL Lipo/miR‐inhibitor; (3) 100 µL Lipo/TGF‐β‐inhibitor; (4) 100 µL Erastin; (5) 100 µL Lipo/Erastin/SB/miR. The treatment process was repeated seven times, and tumor volumes and body weights were measured every three days. Tumor volume was calculated using the formula V = (tumor  length) × (tumor  width)^2^/2.

### 2.14 Immune Cell Profiling by Flow Cytometry

To prepare single‐cell suspensions from xenografted murine PCa tumors, the tumors were digested with a solution containing 1 mg/mL of collagenase IV (Yeason, Shanghai, China) and 5 µg/mL of DNase I (Beyotime, Shanghai, China) in 5 mL of cell culture medium for 1 h. After digestion, cells were stained with a Live/Dead dye (Zombie UV Fixable Viability Kit, BioLegend, San Diego, USA) at a 1:1000 dilution in PBS, incubated on ice for 20 min, and protected from light. Following viability staining, specific antibodies were used to label cell surface markers, and the samples were incubated on ice for an additional 30 min. The stained cells were then analyzed using the BD LSRFortessa X‐20 flow cytometer, with data processed using FlowJo software.

### 2.15 Flow Cytometry Analysis of T Cell‐Mediated Tumor Killing

Tumor cells were seeded in a 12‐well plate at a density of 1×10⁵ cells per 1.5 mL and allowed to adhere. T cells were then added at an effector‐to‐target ratio of 1:1, bringing the total volume to 2.5 mL. After 24 h, effector cells were harvested by washing with PBS, followed by centrifugation at 1800 rpm for 5 min to collect the cell pellet. RM‐1 cells were isolated by centrifugation at 800 rpm for 5 min. The cells were resuspended in 50 µL PBS per tube, and surface marker CD3 on T cells was labeled using an APC‐conjugated antibody in 50 µL PBS. GFP expression was used to assess the proportion of remaining tumor cells. Following a 30 min staining period at 4°C, cells were resuspended in 1 mL of PBS, centrifuged, and the pellet was fixed in 1% paraformaldehyde for flow cytometry analysis.

### 2.16 Statistical Analysis

Except for the mouse experiments, all experiments were conducted with at least three independent biological replicates. Clinical data were sourced from the TCGA database, and subsequent heat maps and volcano plots were generated using the heatmap package in R (https://cran.rproject.org). For survival analysis, log2‐transformed normalized data (log2(normalized value + 1)) were employed, with progression‐free interval (PFI) being assessed using the R survival package. Statistical analyses were performed using GraphPad Prism 9.0 (GraphPad Software, San Diego, USA) and SPSS software version 26.0 (SPSS Inc., Chicago, USA). For normally distributed data, unpaired or paired two‐tailed Student's t‐tests were used to compare the significance of differences between two independent samples. One‐way analysis of variance (ANOVA) was used for comparisons among multiple groups. Prognostic significance was evaluated using Kaplan‐Meier curves and log‐rank tests. Data were presented as the mean ± standard deviation (SD). All experiments were conducted in triplicate, and the average values were used for further analysis. A *p*‐value of less than 0.05 was considered statistically significant.

## 3 Results

### 3.1 Docetaxel‐Resistant CRPC Exhibiting a Positive Correlation With Tumor Cell Stemness

**FIGURE 1:**
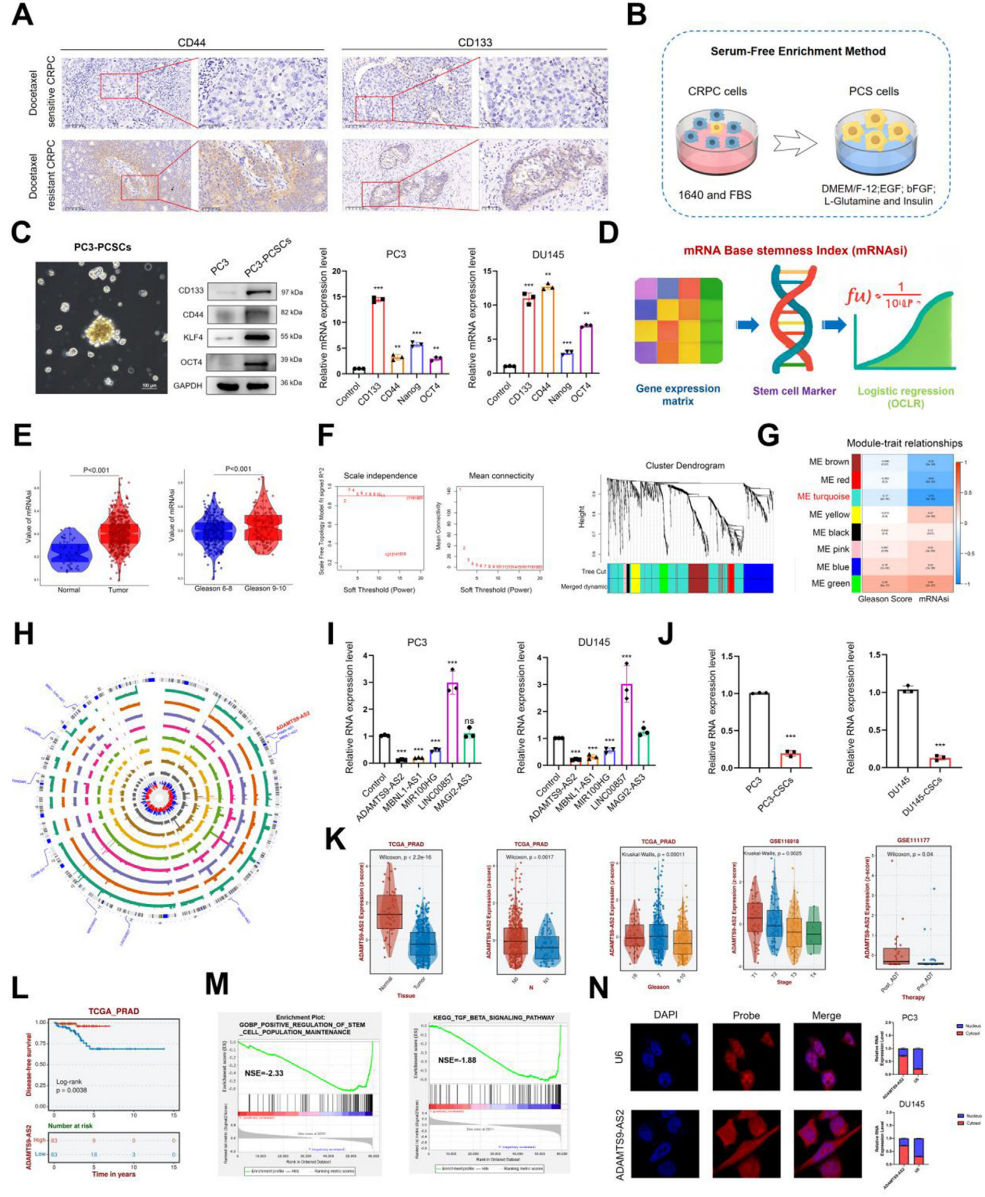
ADAMTS9‐AS2 suppressing Cancer Stemness and Tumor Aggressiveness in DTX CRPC. (A) IHC analysis showing a significant upregulation of CD133 and CD44 in DTX CRPC samples compared to the control group. (B) Workflow of the serum‐free enrichment method for PCSCs isolation. (C) Spheroid formation, WB, and RT‐qPCR assays confirming successful enrichment of PCSCs, evidenced by spheroid formation and expression of stem cell markers. (D) Schematic for the construction of mRNAsi. (E) Box plot showing an increase in mRNAsi in tumor samples, with a significant positive correlation between mRNAsi and Gleason score. (F) Descending order and clustering process of WGCNA. (G) WGCNA results revealing that the turquoise module was significantly negatively correlated with mRNAsi (R = −0.78, p < 0.001) and Gleason score (R = −0.17, p < 0.001). (H) Circos plot illustrating the chromosomal location of ADAMTS9‐AS2. (I) RT‐qPCR results indicating significantly lower expression of ADAMTS9‐AS2 in CRPC cell lines. (J) RT‐qPCR results showing a significant downregulation of ADAMTS9‐AS2 in CRPC‐derived PCSCs. (K) Box plot results demonstrating that ADAMTS9‐AS2 was significantly downregulated in tumor samples from the TCGA‐PRAD cohort, with a negative correlation with N stage, Gleason score, and stage, and an increase after ADT treatment. (L) K‐M survival analysis revealing a significant negative correlation between ADAMTS9‐AS2 expression and overall survival in PCa patients. (M) GSEA based on TCGA‐CRPC samples suggesting that ADAMTS9‐AS2 might be involved in regulating stemness maintenance and the TGF‐β signaling pathway. (N) FISH results showing that ADAMTS9‐AS2 was expressed in both the nucleus and cytoplasm of CRPC cells. Results were presented as mean ± SD. ns indicated *p* > 0.05; ^*^ indicated *p* < 0.05; ^**^ indicated *p* < 0.01; ^***^ indicated *p* < 0.001.

First, a comparison of immunohistochemical results from docetaxel‐resistant and docetaxel‐sensitive CRPC patient tissues was conducted. The results revealed that the expression of PCSC markers CD133 and CD44 was significantly elevated in the tumor tissues of the docetaxel‐resistant group (Figure 1A). Based on RT‐qPCR results from 10 paired docetaxel‐sensitive and resistant CRPC samples, it was shown that the mRNA levels of CD133 and CD44 were significantly increased in the docetaxel‐resistant group (Figure 1A and Figure S1A), suggesting that patients developing docetaxel resistance in CRPC showed increased intratumoral PCSCs abundance, indicating a potential mechanism for therapeutic failure. Using a serum‐free enrichment method (Figure 1B), prostate cancer stem cells were obtained from the CRPC cell line (PC3) and validated by sphere formation, RT‐qPCR, and western blotting. It was found that PC3 cells, after serum‐free enrichment, were capable of forming spheres, and the RNA and protein expression levels of stem cell markers CD133, CD44, Nanog, and OCT4 were elevated (Figure 1C).

Subsequently, using the OCLR algorithm developed by T. M. Malta, et al. [15], based on the gene expression matrix from the TCGA‐PRAD cohort, the stemness index (mRNAsi) was calculated for 499 PCa patients (Figure 1D). Further clinical sample validation of mRNAsi revealed that mRNAsi was significantly elevated in tumor patients, mRNAsi had a significant positive correlation with the Gleason score that was mRNAsi increased with higher tumor differentiation grades (Figure 1E), and mRNAsi had a strong negative correlation with progression‐free interval (PFI) (Figure S1B). Differential gene expression analysis of the high and low mRNAsi groups, followed by GO analysis, indicated that mRNAsi was significantly associated with stem cell proliferation, division, and differentiation (Figure S1C,D). KEGG pathway analysis revealed that mRNAsi was significantly correlated with prostate cancer‐related pathways as well as known stemness‐associated pathways, such as the Hippo, Wnt, and TGF‐beta pathways (Figure S1E). These results suggested that mRNAsi could effectively assess the stemness capacity of prostate cancer samples.

Next, WGCNA based on the TCGA‐PRAD cohort was performed to explore the gene module most strongly correlated with post‐chemotherapy recurrence through mRNAsi screening after dimensional reduction and clustering (Figure 1F). The results showed that genes in the ME turquoise module were significantly negatively correlated with both stemness and malignant differentiation (Figure 1G). The top 10 genes in this module were further examined for their chromosomal localization (Figure 1H), and RT‐qPCR was used to validate the expression of the top 5 genes in CRPC cell lines (PC3 and DU145) (Figure 1I). The results indicated that LncRNA ADAMTS9‐AS2 was significantly downregulated in CRPC cells (Figure 1I). As expected, it was also significantly downregulated in PCSCs derived from CRPC compared to CRPC cells (Figure 1J).

Clinical feature analysis based on public PRAD cohorts revealed that ADAMTS9‐AS2 was significantly underexpressed in tumor samples and was negatively correlated with clinical features associated with malignant progression, including tumor node metastasis, T stage, and Gleason score (Figure 1K). Furthermore, ADAMTS9‐AS2 expression increased after ADT, but was significantly reduced in patients with radiotherapy recurrence (Figure 1K and Figure S1H) and was significantly negatively correlated with PFI (Figure 1L). Subsequent Gene Set Enrichment Analysis (GSEA) using the TCGA‐CRPC cohort indicated that ADAMTS9‐AS2 might play a role in maintaining stemness and was involved in TGF‐beta and Hedgehog signaling pathways (Figure 1M, Figure S1F,G). Further FISH analysis revealed that ADAMTS9‐AS2 was expressed both in the cytoplasm and the nucleus, with the majority of expression localized to the cytoplasm (Figure 1N). These findings suggested that ADAMTS9‐AS2 might regulate PCSCs and inhibit the malignant progression of CRPC.

### 3.2 ADAMTS9‐AS2 Inhibiting Stemness and Enhancing Sensitivity of CRPC Cells to Docetaxel Therapy

**FIGURE 2:**
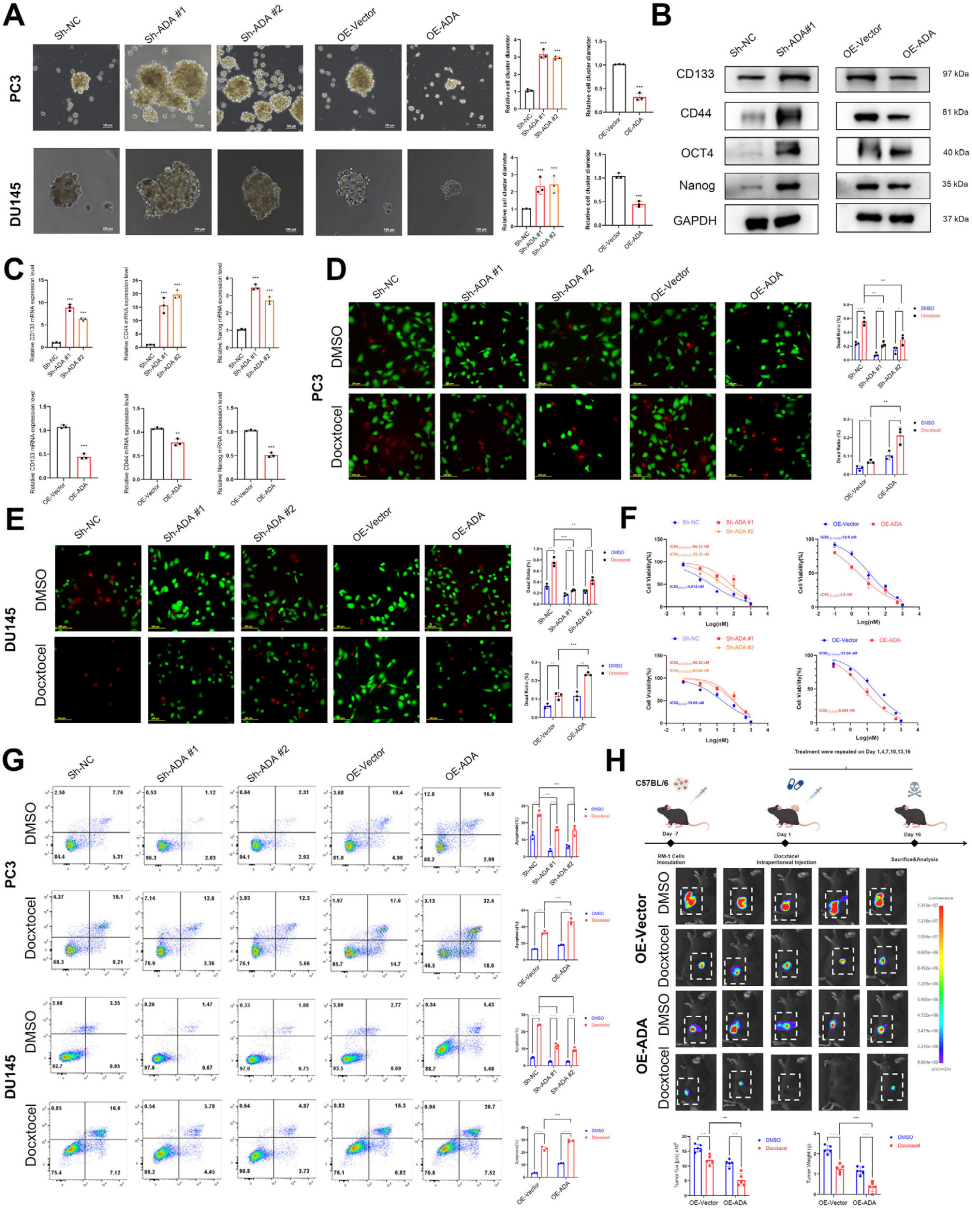
Impact of ADAMTS9‐AS2 on Stemness and Docetaxel Sensitivity in CRPC Cells. (A), (B), and (C) Effects of ADAMTS9‐AS2 expression on stemness in CRPC cell lines, as demonstrated by sphere formation, WB, and RT‐qPCR analyses. (D) and (E) Effect of ADAMTS9‐AS2 expression on docetaxel‐induced cytotoxicity in CRPC cells, as assessed by live/dead staining (red for dead cells, green for live cells). (F) CRPC cells with altered ADAMTS9‐AS2 expression were treated with varying doses of DTX for 48 h, and cell viability was assessed using CCK‐8 assays. (G) Flow cytometry analysis showing the impact of ADAMTS9‐AS2 expression on docetaxel‐induced apoptosis in CRPC cells. (H) Subcutaneous tumor formation in C57BL/6 mice with xenografted RM‐1 overexpressing human ADAMTS9‐AS2, evaluating the impact on docetaxel therapy sensitivity. Results were expressed as mean ± SD. ns indicated *p* > 0.05; ^*^ indicated *p* < 0.05; ^**^ indicated *p* < 0.01; ^***^ indicated *p* < 0.001.

To investigate the effect of ADAMTS9‐AS2 on the stemness of CRPC cells and docetaxel resistance, PC3 and DU145 cells were first transfected with ADAMTS9‐AS2 knockdown and overexpression constructs, and the efficiency was verified by RT‐qPCR (Figure S1I). Subsequently, stemness sphere formation assays were performed to explore the changes in stemness ability after ADAMTS9‐AS2 intervention. The results indicated that knocking down ADAMTS9‐AS2 significantly enhanced the sphere‐forming ability of PCSCs (Figure 2A), with a marked upregulation of stemness‐related markers CD133, CD44, Nanog, and OCT4 at both the mRNA and protein levels (Figure 2B,C). In contrast, overexpression of ADAMTS9‐AS2 significantly reduced sphere formation (Figure 2A) and the expression levels of CD133, CD44, Nanog, and OCT4 (Figure 2B,C), suggesting that ADAMTS9‐AS2 inhibited the stemness capacity of PCSCs.

Furthermore, cell death rate measured by live/dead staining followed by fluorescence microscopy, dose‐dependent cytotoxicity (the half‐maximal inhibitory concentration (IC50)) validated by CCK‐8 proliferation assays, and apoptosis experiments were conducted in vitro to assess the impact of ADAMTS9‐AS2 on the sensitivity of CRPC cell lines to docetaxel treatment. The results indicated that overexpression of ADAMTS9‐AS2 led to increased CRPC cell death (Figure 2D,E), a decrease in IC50 value (Figure 2F), and an increase ratio in apoptosis (Figure 2G) upon docetaxel treatment. On the contrary, ADAMTS9‐AS2 depletion caused decreased CRPC cell death (Figure 2D,E), an increase in IC50 value (Figure 2F), and a decrease ratio in apoptosis (Figure 2G) after docetaxel treatment.

To further confirm these findings in vivo, a RM‐1 cell line stably over‐expressing ADAMTS9‐AS2 was established (Figure S2A) and subcutaneously injected into C57BL/6 mice. After seven days, docetaxel treatment was initiated with intraperitoneal injections every three days for a total of six treatments. The results showed that the subcutaneous tumors in the overexpression group were significantly smaller, indicating enhanced sensitivity to docetaxel treatment (Figure 2H).

These results suggested that ADAMTS9‐AS2 might enhance the sensitivity of CRPC cells to docetaxel therapy by inhibiting the stemness of PCSCs, thus delaying the onset of resistance.

### 3.3 ADAMTS9‐AS2 Reversing Docetaxel Resistance and Inhibiting Malignant Progression in CRPC Cells

**FIGURE 3:**
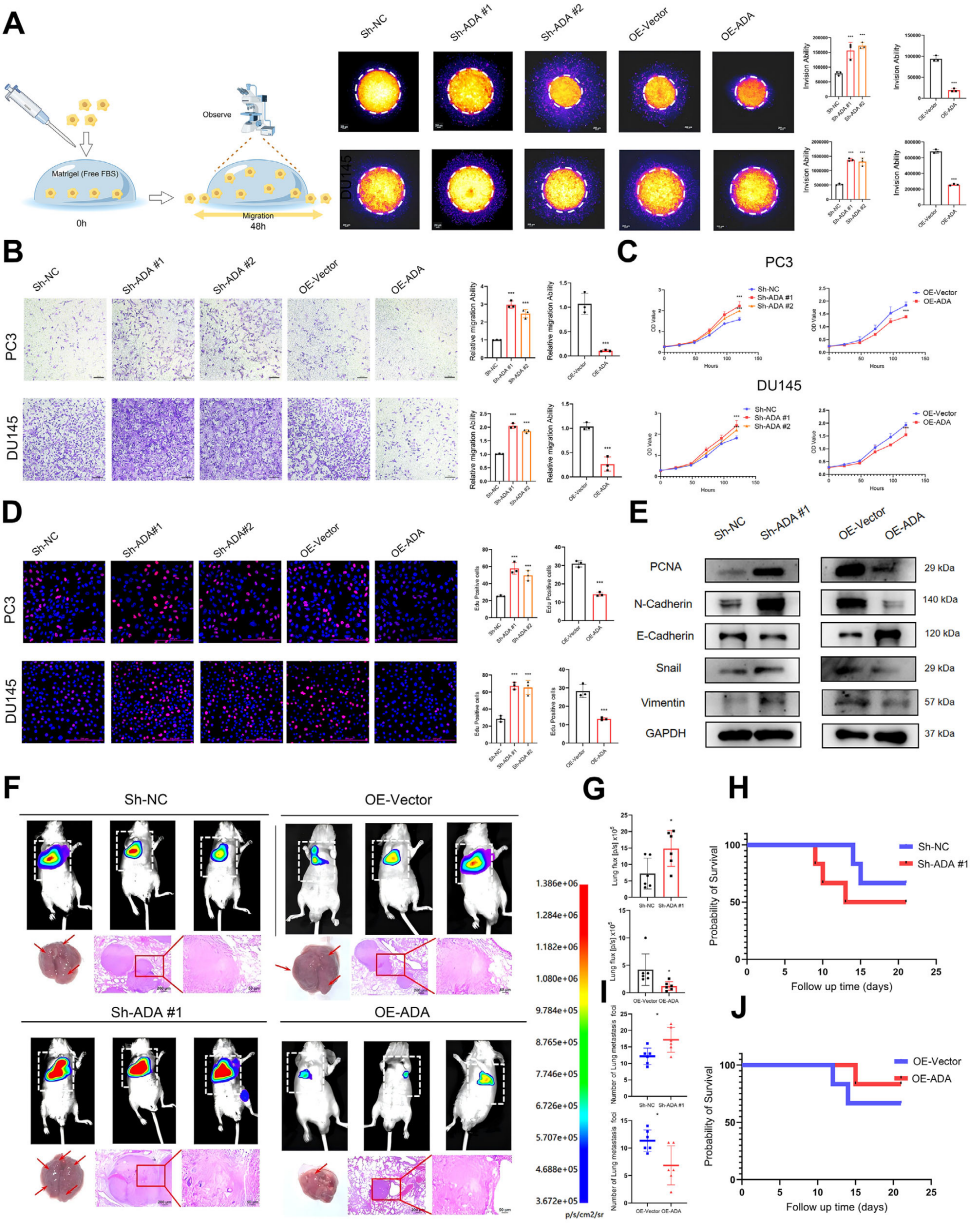
Impact of ADAMTS9‐AS2 on Docetaxel Resistance and Malignant Progression in CRPC Cells. (A) and (B) 3D invasion and transwell assays demonstrating the impact of ADAMTS9‐AS2 expression on the invasion and migration ability of DTX CRPC cells under 20 nM docetaxel treatment. (C) and (D) CCK‐8 and EdU assays demonstrating the impact of ADAMTS9‐AS2 expression on the proliferation of DTX CRPC cells under 20 nM docetaxel treatment. (E) WB analysis used to further validate the expression changes of EMT and proliferation markers after modulation of ADAMTS9‐AS2 expression. (F‐J) Effect of ADAMTS9‐AS2 expression on the in vivo invasion ability of DU145 cells, evaluated by mouse lung metastasis assay. KM survival curves showing the survival of nude mice after lung metastasis. Results were expressed as mean ± SD. ns indicated *p* > 0.05; ^*^ indicated *p* < 0.05; ^**^ indicated *p* < 0.01; ^***^ indicated *p* < 0.001.

To further investigate whether ADAMTS9‐AS2 could reverse docetaxel resistance, docetaxel‐resistant CRPC cell lines (with 10 times of the baseline IC50) were established and maintained the culture with 10 nM docetaxel in both PC3 and DU145 (Figure S2B,C). Subsequently, 3D invasion, Transwell, and wound healing assays were performed to assess whether ADAMTS9‐AS2 affected the invasion and migration abilities of the drug‐resistant cells (Figure 3A,B; Figure S2D,E). CCK‐8 and EdU assays were used to determine whether ADAMTS9‐AS2 influenced the proliferation capacity of the drug‐resistant cells (Figure 3C,D). The results indicated that knockdown of ADAMTS9‐AS2 significantly promoted the number of cell invasions and migrations, the rate of cell wound healing, the cell proliferation abilities, and the number of EdU‐positive cells in docetaxel‐resistant CRPC cells in vitro, while overexpression of ADAMTS9‐AS2 had the opposite effect (Figure 3A‐D; Figure S2D,E). Western blotting further confirmed the changes in cell proliferation and invasion‐related markers after ADAMTS9‐AS2 knockdown or overexpression, supporting the aforementioned conclusions (Figure 3E). Notably, knockdown of ADAMTS9‐AS2 significantly enhanced the expression of the proliferation marker PCNA, along with upregulation of mesenchymal markers Vimentin and N‐Cadherin, concomitant with downregulation of the epithelial marker E‐Cadherin, collectively indicating the induction of epithelial‐mesenchymal transition (EMT). Conversely, overexpression of ADAMTS9‐AS2 resulted in reduced PCNA expression, suppression of Vimentin and N‐Cadherin, and restoration of E‐Cadherin levels (Figure 3E).

To further validate the effect of ADAMTS9‐AS2 on the docetaxel‐resistant cell line in vivo, a mouse lung metastasis model was used. Mice were injected via the tail vein to establish the model, and docetaxel treatment was initiated after 7 days with intraperitoneal injections every three days. The results indicated that knockdown of ADAMTS9‐AS2 significantly increased the number and size of lung and intestinal metastases and reduced the survival time of the mice. In contrast, overexpression of ADAMTS9‐AS2 significantly reduced the number and size of lung and intestinal metastases, and increased the survival time of the mice (Figure 3F–J; Figure S2F,G). Immunohistochemical analysis of the metastasis‐related marker N‐Cadherin further supported the role of ADAMTS9‐AS2 in promoting the metastatic ability of docetaxel‐resistant CRPC cells (Figure S2H). Notably, ADAMTS9‐AS2 knockdown upregulated N‐cadherin in pulmonary metastases, while ADAMTS9‐AS2 overexpression had the opposite effect, suppressing its expression (Figure S2H). These results suggested that ADAMTS9‐AS2 could reverse the malignant progression of docetaxel‐resistant CRPC cells both in vitro and in vivo.

### 3.4 ADAMTS9‐AS2 Orchestrating Stemness Maintenance and Docetaxel Resistance in CRPC Through FOXF2/TGF‐β Axis

**FIGURE 4:**
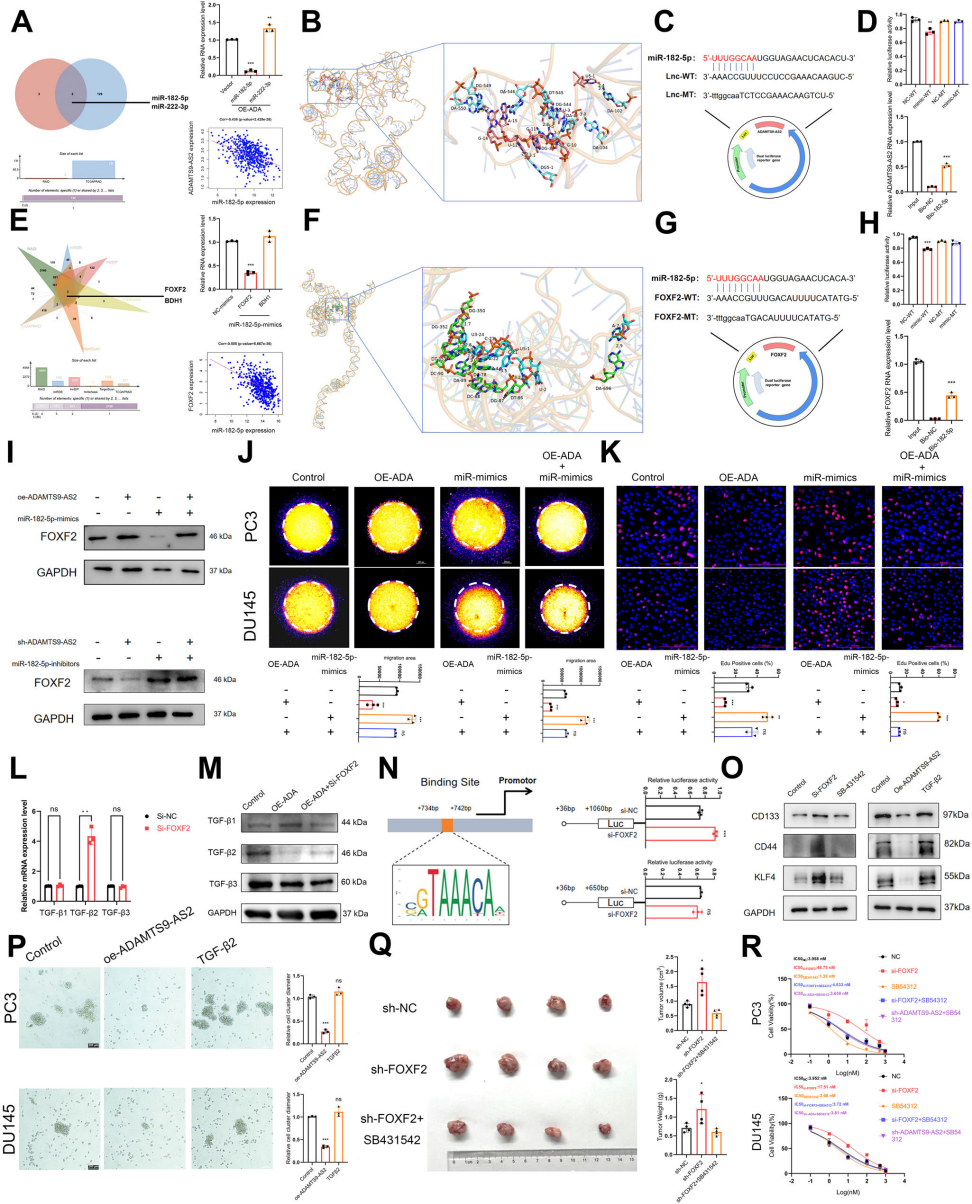
Effect of ADAMTS9‐AS2 on CRPC Stemness and Docetaxel Resistance Mediated by the FOXF2/TGF‐β Axis. (A) Venn plots illustrating downstream microRNAs predicted by RAID, along with correlation analysis of TCGA‐PRAD, and RT‐qPCR validation of microRNAs regulated by ADAMTS9‐AS2. (B) 3D structure prediction showing the binding pattern of ADAMTS9‐AS2 to miR‐182‐5p. (C) and (D) Dual luciferase reporter assay used to detect the binding of ADAMTS9‐AS2 to miR‐182‐5p, further supported by RNA‐pulldown experiments. (E) Venn plot showing the combination of miRDB, miRDIP, miRTarBase, RAID, and TargetScan, with mRNAs predicted downstream by the TCGA database, and RT‐qPCR validation of mRNAs regulated by miR‐182‐5p. (F) 3D structure prediction showing the binding pattern of FOXF2 to miR‐182‐5p. (G) and (H) Dual luciferase reporter assay used to detect the binding of ADAMTS9‐AS2 to miR‐182‐5p, further supported by RNA‐pulldown experiments. (I) WB demonstrating the correlation between ADAMTS9‐AS2, miR‐182‐5p, and FOXF2. (J) and (K) Transwell and EdU assays demonstrating that ADAMTS9‐AS2 regulating the invasion and proliferation of DTX CRPC cells through the miR‐182‐5p/FOXF2 pathway. (L) and (M) RT‐qPCR and WB showing altered TGF‐β family mRNA and protein levels after FOXF2 knockdown. (N) Schematic representation of the TGF‐β2 promoter sequence and identification of the FOXF2‐binding ability by dual‐luciferase reporter assay. (O) and (P) WB and sphere formation assays evaluating whether SB54312 rescued the induction of stemness in CRPC cells caused by FOXF2 knockdown. (Q) Subcutaneous xenograft assay evaluating whether SB54312 rescued the enhanced proliferative capacity of CRPC cells induced by FOXF2 knockdown. (R) CCK‐8 assay assessing whether SB54312 rescued the reduced docetaxel sensitivity of CRPC cells caused by FOXF2 knockdown. Results were presented as mean ± SD. ns indicated *p* > 0.05; ^*^ indicated *p* < 0.05; ^**^ indicated *p* < 0.01; ^***^ indicated *p* < 0.001.

To further elucidate the molecular mechanism by which ADAMTS9‐AS2 exerted its function, bioinformatics analysis based on public databases combined with RT‐qPCR results from ADAMTS9‐AS2 overexpression revealed that ADAMTS9‐AS2 could bind to miR‐182‐5p (Figure 4A). Through integrated analysis of the RNA‐associated Interaction Database (RAID) and TCGA PRAD, we identified potential microRNAs capable of binding ADAMTS9‐AS2. Intersection of these two independent predictions revealed only two candidates: miR‐182‐5p and miR‐222‐3p. Subsequent functional experiments demonstrated that ADAMTS9‐AS2 overexpression led to decreased miR‐182‐5p levels but increased miR‐222‐3p expression. This inverse correlation between ADAMTS9‐AS2 and miR‐182‐5p was further supported by analysis of TCGA prostate cancer data, suggesting potential ADAMTS9‐AS2 degradation mediated by miR‐182‐5p (Figure 4A). Based on these consistent findings, we selected miR‐182‐5p for further mechanistic investigation. Survival analysis showed that miR‐182‐5p was significantly negatively correlated with the prognosis of PCa patients (Figure S3A). Clinical correlation analysis revealed that miR‐182‐5p was significantly positively correlated with high TNM stage (Figure S3B–D), recurrence (Figure S3E), and high Gleason score (Figure S3F) in PCa.

Further validation through 3D molecular docking, dual‐luciferase reporter assays, and RNA‐pulldown experiments confirmed these findings (Figure 4B–D). In silico analysis, including 3D molecular docking and bioinformatics prediction, suggested that ADAMTS9‐AS2 harbored a sequence complementary to the 5′ seed region of miR‐182‐5p, indicative of a potential binding interaction (Figure 4B,C). To experimentally validate this, we cloned wild‐type (WT) and mutant (Mut) fragments of ADAMTS9‐AS2—containing either the intact or disrupted miR‐182‐5p binding sequence, respectively—into a dual‐luciferase reporter vector (Figure 4C). Upon co‐transfection with miR‐182‐5p mimics, the WT ADAMTS9‐AS2 construct exhibited a significant reduction in luciferase activity, whereas the Mut ADAMTS9‐AS2 showed no appreciable change (Figure 4D). To further validate the interaction between miR‐182‐5p and ADAMTS9‐AS2, we performed an RNA pull‐down assay using in vitro‐synthesized biotinylated miR‐182‐5p mimics, with biotinylated scramble mimics serving as a negative control. Following incubation with cell lysates, streptavidin beads capture, and extensive washing, the pulled‐down RNAs were analyzed by RT‐qPCR. Notably, ADAMTS9‐AS2 was significantly enriched in the miR‐182‐5p mimic pull‐down fraction compared to the scramble control (*p* < 0.001), providing direct biochemical evidence for the specific association between miR‐182‐5p and ADAMTS9‐AS2 (Figure 4D). In conclusion, these results confirmed a direct interaction between miR‐182‐5p and ADAMTS9‐AS2, mediated by the predicted seed‐pairing sequence.

Additionally, analysis from six databases, combined with molecular experiments, further confirmed that miR‐182‐5p could bind to FOXF2 (Figure 4E–H). Through integrated analysis of the RAID database, MicroRNA Target Prediction Database (miRDB), microRNA Data Integration Portal (mirDIP), miRBase, TargetScan, and TCGA PRAD datasets, we identified potential gene targets of miR‐182‐5p. The intersection of these six independent predictions revealed only two candidates: FOXF2 and BDH1. Subsequent functional experiments demonstrated that miR‐182‐5p overexpression led to decreased FOXF2 levels but unchangeable BDH1 expression. This negative correlation between miR‐182‐5p and FOXF2 was further supported by analysis of TCGA prostate cancer data, suggesting potential FOXF2 degradation mediated by miR‐182‐5p (Figure 4E).

In silico analysis, including 3D molecular docking and bioinformatics prediction, suggested that FOXF2 harbored a sequence complementary to the 5′ seed region of miR‐182‐5p, indicative of a potential binding interaction (Figure 4F,G). To experimentally validate this, we cloned WT and Mut fragments of FOXF2—containing either the intact or disrupted miR‐182‐5p binding sequence, respectively—into a dual‐luciferase reporter vector (Figure 4G). Upon co‐transfection with miR‐182‐5p mimics, the WT FOXF2 construct exhibited a significant reduction in luciferase activity, whereas the Mut FOXF2 showed no appreciable change (Figure 4H). To further validate the interaction between miR‐182‐5p and FOXF2, we performed an RNA pull‐down assay using in vitro‐synthesized biotinylated miR‐182‐5p mimics, with biotinylated scramble mimics serving as a negative control. Following incubation with cell lysates, streptavidin beads capture, and extensive washing, the pulled‐down RNAs were analyzed by RT‐qPCR. Notably, FOXF2 was significantly enriched in the miR‐182‐5p mimic pull‐down fraction compared to the scramble control (p < 0.001), providing direct biochemical evidence for the specific association between miR‐182‐5p and FOXF2 (Figure 4H). In conclusion, these results confirmed a direct interaction between miR‐182‐5p and FOXF2, mediated by the predicted seed‐pairing sequence, as the same as the interaction between miR‐182‐5p and ADAMTS9‐AS2.

Survival analysis showed that FOXF2 was significantly positively correlated with the prognosis of PCa patients (Figure S3G), and was significantly downregulated in tumor samples (Figure S3H). IHC results from clinical samples also indicated that FOXF2 was significantly downregulated in tumor samples (Figure S3M). Clinical correlation analysis revealed that FOXF2 expression was significantly negatively correlated with high N stage (Figure S3I) and high biochemical recurrence (Figure S3J) in PCa. Further functional assays, including EdU and Transwell experiments, demonstrated the tumor‐suppressive and oncogenic impact of FOXF2 and miR‐182‐5p on the malignant progression of CRPC, respectively. The results indicated that knocking down FOXF2 or overexpressing miR‐182‐5p significantly promoted the rate of DNA synthesis and the number of cell migration in both DU145 and PC3 cells (Figure S4A–C).

Western blotting results showed that overexpression of ADAMTS9‐AS2 could restore the decreased FOXF2 expression caused by overexpression of miR‐182‐5p (Figure 4I). Additionally, knockdown of ADAMTS9‐AS2 could restore the increased FOXF2 expression induced by suppression of miR‐182‐5p (Figure 4I). Furthermore, 3D Transwell and EdU assays at the cellular functional level further supported the notion that the ADAMTS9‐AS2/miR‐182‐5p/FOXF2 axis could inhibit the proliferation and invasion in docetaxel‐resistant CRPC cells (Figure 4J,K). ADAMTS9‐AS2 overexpression could restore the increased ability of cell invasion and the increased number of EdU‐positive cells caused by miR‐182‐5p overexpression (Figure 4J,K).

To further clarify the downstream mechanisms by which this axis exerted its function, GSEA enrichment analysis revealed that FOXF2 was involved in regulating the TGF‐β pathway, which overlapped with the pathway involved in ADAMTS9‐AS2 (Figure S3K,L). It was hypothesized that FOXF2 might regulate the expression of TGF‐β as a transcription factor. After knocking down FOXF2, it was found that among the three transforming growth factors (TGF‐β1, TGF‐β2, TGF‐β3) involved in the TGF‐β pathway, only TGF‐β2 was significantly upregulated at both the transcriptional and protein levels, suggesting a specific regulatory role of FOXF2 in the TGF‐β pathway in prostate cancer (Figure 4L,M). Further, using the JASPAR database, we predicted the binding site of FOXF2 in the TGF‐β2 promoter region and successfully constructed dual‐luciferase reporter plasmids with and without the FOXF2 binding site (734–742 bp) in the TGF‐β2 promoter region. After transfecting the plasmids, we observed an increase in fluorescence intensity in the plasmids containing the binding site upon FOXF2 depletion, while no change was observed in the plasmids lacking the binding site, indicating that FOXF2 could bind to the promoter region of TGF‐β2 and exert a transcriptional repression function (Figure 4N).

Western blotting results further suggested that using a TGF‐β inhibitor (SB‐431542) could restore the elevated stemness markers (CD133, CD44, and KLF4) caused by FOXF2 knockdown, while adding TGF‐β2 rescued the stemness marker reduction caused by ADAMTS9‐AS2 overexpression (Figure 4O). Sphere formation assays further supported these changes at the stem cell morphology level, adding TGF‐β2 could rescue the reduction of stem cell number and diameter caused by ADAMTS9‐AS2 overexpression (Figure 4P). IHC results further supported the positive regulatory relationship between ADAMTS9‐AS2 and FOXF2 (Figure S4E). In vivo experiments showed that SB431542 could reverse the fast tumor growth rate and upregulation of stemness markers caused by sh‐FOXF2 (Figure 4Q, Figure S4D,F). After treating CRPC cell lines with docetaxel, we found that knocking down FOXF2 increased the IC50. When docetaxel was combined with SB431542, the IC50 decreased; however, knocking down FOXF2 and supplying with SB431542 did not change the IC50, suggesting that the abolished transcriptional repression of TGF‐β2 by FOXF2 depletion was restored by SB431542. Consistent with this, knocking down ADAMTS9‐AS2 in combination with SB431542 did not change the IC50 (Figure 4R).

In conclusion, these results suggested that ADAMTS9‐AS2 negatively regulated PCSC self‐renewal through the FOXF2/TGF‐β axis, ultimately inhibiting docetaxel resistance in prostate cancer.

### 3.5 ADAMTS9‐AS2 Encoding a Short Peptide Which Regulating Ferroptosis and Docetaxel Resistance in CRPC via Binding to SLC7A11

**FIGURE 5:**
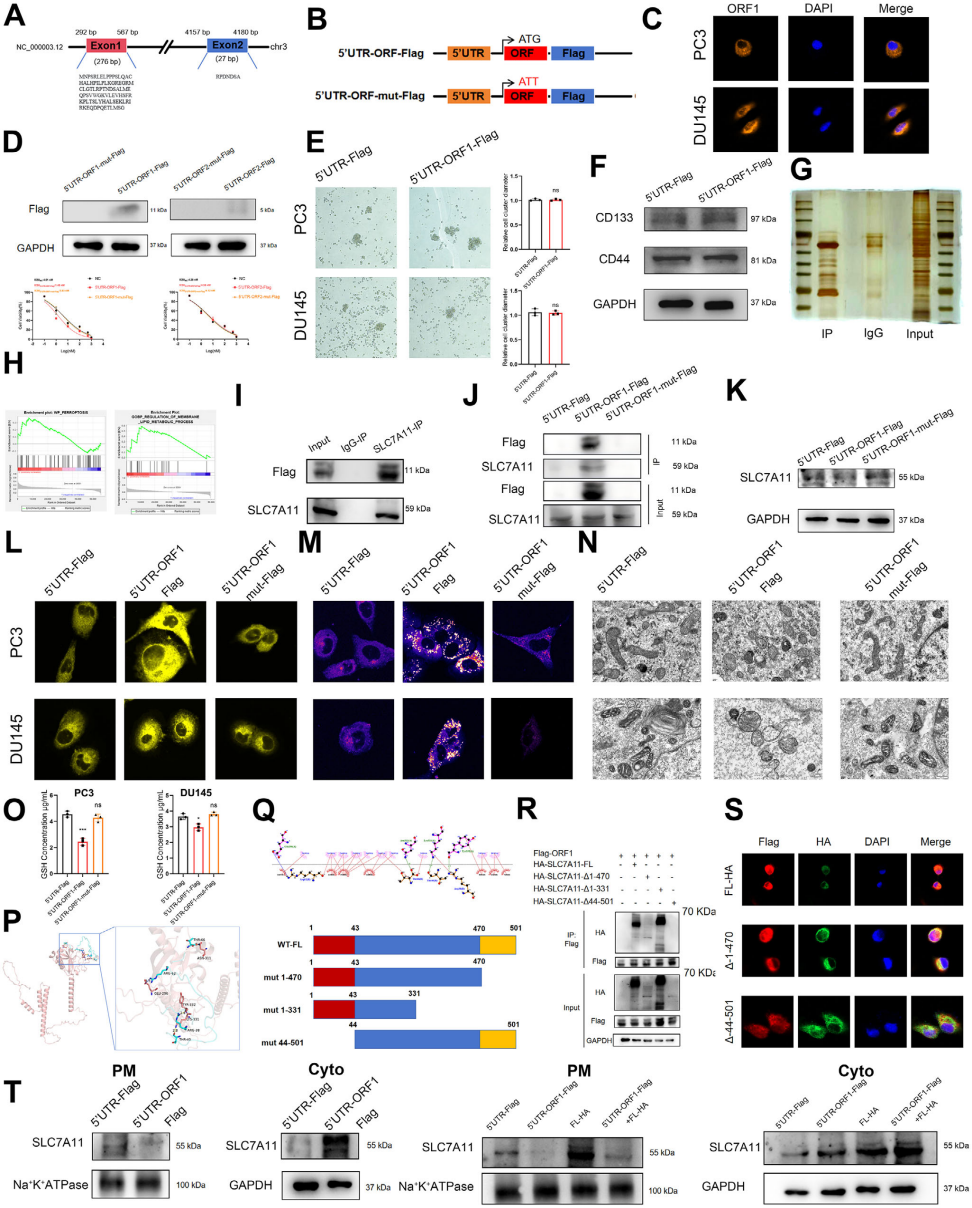
ADAMTS9‐AS2 Encoding a Short Peptide that Regulating Ferroptosis and Docetaxel Resistance in CRPC through SLC7A11 Binding. (A) Position and sequence of the two ORFs in ADAMTS9‐AS2. (B) Construction of a plasmid containing the ADAMTS9‐AS2 open reading frame sequences. (C) Immunofluorescence analysis showing the cellular localization of ORF1. (D) WB analysis of ORF1 expression in CRPC cells and CCK‐8 assay assessing the impact of ORFs on DTX sensitivity in CRPC cells. (E) and (F) Sphere formation and WB assays evaluating the effect of ORF1 overexpression on stemness in CRPC cells. (G) and (H) Co‐IP followed by mass spectrometry identification of proteins interacting with ORF1 and their associated biological processes. (I) and (J) Co‐IP followed by WB analysis to verify the interaction between the ORF1‐encoded peptide and SLC7A11. (K) WB analysis to verify the effect of ORF1 overexpression on SLC7A11 expression. (L‐O) Ferrous ion detection, ROS detection, cell electron microscopy, and GSH assays to validate the changes in ferroptosis levels in CRPC cells after ORF1 overexpression. (P) 3D molecular structure simulation of short peptide 1 binding to the SLC7A11 binding site. (Q‐R) Design of a mutant plasmid targeting the functional region of SLC7A11 and verification of the binding site between short peptide 1 and SLC7A11 through IP experiments. (S) Immunofluorescence experiment exploring the co‐localization of short peptide 1 with the mutant SLC7A11 protein. (T) WB analysis used to verify the expression changes of SLC7A11 in the cytoplasm and cell membrane following overexpression of short peptide 1. Results were presented as mean ± SD. ns indicated *p* > 0.05; ^*^ indicated *p* < 0.05; ^**^ indicated *p* < 0.01; ^***^ indicated *p* < 0.001.

The FISH results indicated that ADAMTS9‐AS2 was not only present in the cytoplasm but also expressed in the nucleus. In recent years, with the advancement of genomics technologies, increasing evidence had suggested that LncRNAs not only play roles in transcriptional regulation but also had potential coding functions, being able to translate into small functional peptides. To further investigate whether ADAMTS9‐AS2 exerted its function in the nucleus, bioinformatics analysis based on public databases revealed that ADAMTS9‐AS2 contained two open reading frames (ORFs), located at 292–567 and 4157–4180 bp, respectively (Figure 5A), suggesting its potential ability to encode short peptides. Based on the amino acid sequence, we constructed 2D structure models for the two short peptides (Figure S4G,H). Subsequently, we constructed wild‐type and the start codon mutated (ATG>ATT) Flag‐tagged recombinant plasmids containing the ORFs (Figure 5B) and validated their localization (both in cytoplasma and nuclear) and expression at the cell level (Figure 5C,D). CCK‐8 assays further demonstrated that the short peptide 1 encoded by the ORF1 sequence had the ability to inhibit the cell viability upon docetaxel treatment in docetaxel‐resistant prostate cancer cell lines, while short peptide 2 did not exhibit this ability (Figure 5D). Immunofluorescence results indicated that most of the short peptide 1 was located in the cytoplasm (Figure 5C).

However, further experimental results revealed that short peptide 1 did not affect the sphere‐forming ability of PCSCs or the expression levels of stemness markers (Figure 5E,F), indicating ADAMTS9‐AS2 encoded short peptide 1 didn't affect cell stemness, which meant the short peptide 1 might function at other ways. Therefore, we further performed short peptide 1 pull‐down assay and proteinmic analysis and discovered that this short peptide might affect the ferroptosis process in prostate cancer cells by binding to SLC7A11 (Figure 5G,H). GSEA results also suggested that ADAMTS9‐AS2 might be involved in regulating ferroptosis and membrane lipid metabolic process (Figure 5H). To further clarify whether the short peptide and FOXF2 affect the role of ADAMTS9‐AS2 in ferroptosis in CRPC cells, we conducted additional rescue experiments. Our results demonstrated that overexpression of the short peptide 1 could significantly reverse the inhibition of ferroptosis induced by ADAMTS9‐AS2 knockdown in CRPC cells. However, knockdown of FOXF2 did not affect the ferroptosis induced by overexpression of ADAMTS9‐AS2. These findings suggested that ADAMTS9‐AS2 promoted ferroptosis in CRPC cells via the short peptide, thereby inhibiting docetaxel resistance, and this mechanism was independent of the FOXF2/TGF‐β axis (Figure S4I–K). Moreover, short peptide 1 did not affect autophagy or apoptosis in CRPC cells (Figure S5A). IP assays further validated that short peptide 1 could bind to SLC7A11 (Figure 5I), whereas the mutated short peptide 1 could not bind (Figure 5J). We also found that short peptide 1 did not affect the expression level of SLC7A11 (Figure 5K).

Subsequently, Fe^2+^ staining, ROS probes, mitochondrial electron microscopy, and GSH content assays were performed to investigate the effect of short peptide 1 on ferroptosis in CRPC cells. The results indicated that short peptide 1 promoted Fe^2+^ accumulation, increased ROS levels, elevated mitochondrial collapse, and reduced GSH, thereby promoting ferroptosis in CRPC cells (Figure 5L–O).

To further explore the mechanism by which short peptide 1 regulated ferroptosis through binding to SLC7A11, molecular docking techniques were employed to identify the binding site between short peptide 1 and SLC7A11 (Figure 5P). Based on functional regions, three truncated mutations for SLC7A11 were constructed: 1–470, 1–331, and 44–501 bp (Figure 5Q). IP assay results further suggested all the truncated mutations except 44–501 bp truncation could be pulled down by short peptide 1, indicating that the 1–43 bp region of SLC7A11 might bind to short peptide 1 of ADAMTS9‐AS2 (Figure 5R). Immunofluorescence co‐localization experiments confirmed that short peptide 1 colocalized with the 1–43 bp region of SLC7A11 in the cytoplasm of CRPC cells (Figure 5S and Figure S5B). After performing cytoplasma‐cytomembrane fractionation experiments and further validating via western blotting, we confirmed that overexpression of short peptide 1 reduced the presence of SLC7A11 on the cell membrane and concurrently increased its accumulation in the cytoplasm. Furthermore, co‐overexpression of short peptide 1 and SLC7A11 did not rescue the membrane localization of SLC7A11, despite elevated SLC7A11 expression levels. Instead, SLC7A11 continued to accumulate predominantly in the cytoplasm (Figure 5T). These results indicated that short peptide 1 inhibited the translocation of SLC7A11 to the cell membrane, that is short peptide 1 binded to SLC7A11 in the cytoplasm, leading to a decrease of SLC7A11 on the cell membrane.

In a word, these results suggested that the short peptide 1 translated by ADAMTS9‐AS2 did not regulate PCSCs’ function but instead binded to SLC7A11 in the cytoplasm, causing it to accumulate in the cytoplasm and preventing its cell membrane localization to form the cystine/glutamate antiporter (System Xc ‐). This delayed the influx of GSH and promoted ferroptosis in CRPC cells, ultimately inhibiting docetaxel resistance.

### 3.6 Construction and Evaluation of Targeted Nanomaterials for Inhibiting Stemness and Ferroptosis in Docetaxel‐Resistant CRPC

**FIGURE 6:**
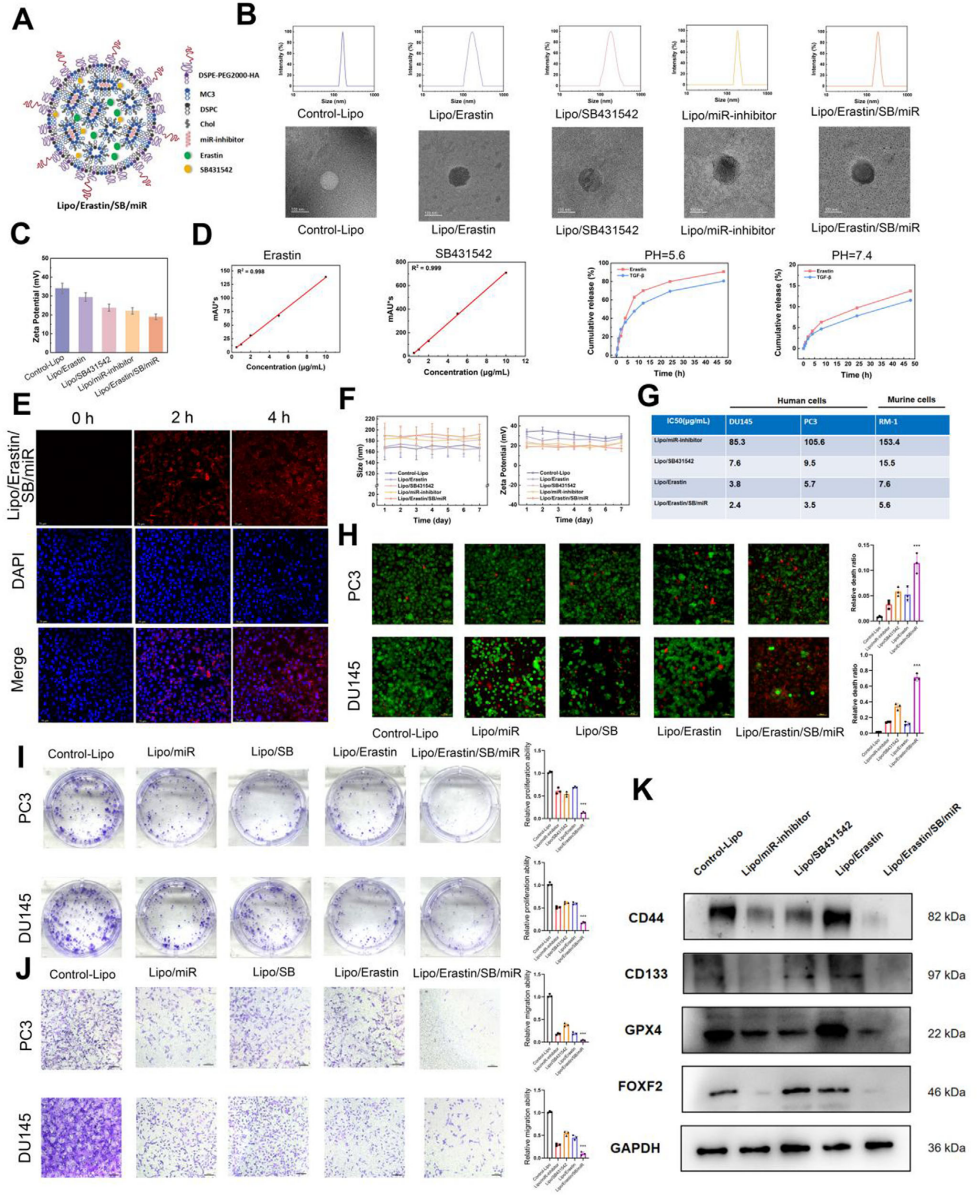
Targeted Nanomaterials for Inhibiting Stemness and Inducing Ferroptosis in DTX CRPC. (A) Schematic representation of the LP structure, illustrating the compartmentalization of Erastin and TGF‐β inhibitor in the hydrophobic layer and miRNA in the hydrophilic layer. (B) The images showing the dynamic light scattering and transmission electron microscopy images of the liposomes. (C) Zeta potential measurements of different liposomes, showing positive surface charges for all formulations, confirming successful preparation of cationic liposomes. The surface potential decreased slightly after incorporation of miRNA and drugs. (D) Calibration curves for quantification of Erastin and TGF‐β inhibitor content in LPs by HPLC. Drug release profiles of LPs under different pH conditions. Both Erastin and TGF‐β inhibitor were released faster at pH 5.6, mimicking the acidic tumor microenvironment, compared to physiological pH. (E) Fluorescence microscopy imaging of nanomaterial uptake in CRPC cells. (F) Liposomes stored at 4°C showing minimal changes in particle size and zeta potential over 7 days, with a uniform size distribution, indicating good stability. (G) IC50 values of the nanomaterials in various cell lines determined using the CCK‐8 assay. (H) The Live/Dead assay used to assess the cytotoxic effect of nanomaterials on DTX‐resistant CRPC cells. (I‐J) Colony formation and Transwell assays used to assess the impact of different nanomaterials on the proliferation and invasion of DTX‐resistant CRPC cells. (K) WB used to evaluate the impact of different nanomaterials on the expression of corresponding downstream targets in CRPC cells. Results were presented as mean ± SD. ns indicated *p* > 0.05; ^*^ indicated *p* < 0.05; ^**^ indicated *p* < 0.01; ^***^ indicated *p* < 0.001.

The cationic liposomes (LPs) were successfully prepared according to the component ratios in Table 1, and the structure was shown in Figure 6A. Due to the different hydrophilic and hydrophobic properties of the components, the ferroptosis inducer (Erastin) and the TGF‐β inhibitor (SB431542) were sequestered within the hydrophobic layer of the liposomes, while the miRNA inhibitor was distributed in the hydrophilic layer. This compartmentalization strategy leveraged the inherent properties of liposomes to accommodate both hydrophobic and hydrophilic agents simultaneously. The size and morphology of the prepared LPs were determined by dynamic light scattering and transmission electron microscopy. As shown in Figure 6B, the LPs exhibited a spherical morphology with diameters of approximately 100–150 nm, LPs loaded with miRNA and drugs were slightly larger than blank LPs, and the size of all LPs remained below 200 nm, favoring prolonged systemic circulation and tumor accumulation. Figure 6C revealed that the zeta potentials of the different liposomes were positive, confirming the successful preparation of cationic liposomes. After incorporation of the drug molecules or miRNA, the surface potential decreased slightly, consistent with the expected reduction in colloidal stability caused by the additional components. The Erastin and TGF‐β inhibitor contents in the LPs were quantified by high‐performance liquid chromatography (HPLC). The corresponding calibration curves were presented in Figure 6D, and the detailed drug‐loading data were listed in Table 2. The drug‐release profiles of the LPs in buffers of different pH values were subsequently measured. As depicted in Figure 6D, both Erastin and the TGF‐β inhibitor were released markedly faster under acidic conditions (pH 5.6) that mimicked the tumor microenvironment than under physiological conditions, facilitating drug accumulation at the tumor site. Collectively, these results indicated that the developed LPs possessed advantageous characteristics for drug delivery.

**TABLE 2:** Encapsulation efficiency and drug loading efficiency of different LPs.

Groups	Erastin	TGF‐β	Erastin+TGF‐β+miRNA‐inhibitor
Encapsulation efficiency (%)	86.41	47.75	81.8/83.15
Drug loading efficiency (%)	5.79	1.19	1.87/1.08

Due to the targeting ability of the nanomaterials, we further used rhodamine‐labeled liposomes and verified their endocytosis in CRPC cells using fluorescence microscopy. The results suggested that the materials were efficiently absorbed and internalized by the cells (Figure 6E). The liposomes were stored alone at 4°C in a refrigerator, and their particle size and zeta potential were monitored for changes over 7 days. As shown in Figure 6F, the particle size and zeta potential of the five types of liposomes showed minimal changes, and the particle size distribution remained uniform, indicating that the liposomes had good stability. Subsequently, we determined the IC50 values of different formulations in PC3, DU145, and mouse‐derived RM‐1 cells (Figure 6G). Among all formulations, the Lipo/Erastin/miR/SB(Liposomal nanoparticles co‐loaded with SB431542, a miR‐182‐5p inhibitor, and Erastin) group exhibited the lowest IC50 value, followed by the Lipo/Erastin group and then the Lipo/SB431542 group. In contrast, the Lipo/miR‐inhibitor group demonstrated a substantially higher IC50, indicating that cells were most sensitive to treatment with the Lipo/Erastin/miR/SB group, suggesting the highest level of cell death and the greatest therapeutic efficacy. Further, we assessed the cytotoxicity of Lipo/Erastin/miR/SB using Calcein AM/PI staining. PC3 and DU145 cells were exposed to different treatment conditions and subsequently stained with Calcein AM/PI. The results showed that the cell death rate in the Lipo/Erastin/miR/SB group was significantly higher than in the other groups (Figure 6H).

Next, we conducted colony formation assays to evaluate the effect of Lipo/Erastin/miR/SB on the proliferation of docetaxel‐resistant CRPC cells. The results indicated that, compared to the control group, the number of cell colonies in the Lipo/Erastin/miR/SB group was significantly reduced (Figure 6I). CRPC metastasis was a major factor in poor prognosis for patients. To further assess the metastasis effects of Lipo/Erastin/miR/SB on docetaxel‐resistant CRPC cells in vitro, Transwell assays were performed to evaluate the inhibition of CRPC metastasis by these nanoparticles. The results showed that the control group had the highest number of migrating cells, while the Lipo/Erastin/miR/SB group had the least (Figure 6J). These experimental results suggested that Lipo/Erastin/miR/SB could significantly inhibit the migration of docetaxel‐resistant CRPC cells. The above experimental results showed that Lipo/Erastin/miR/SB exhibited good inhibitory effects on the proliferation and metastasis of docetaxel‐resistant CRPC cells. To further detect the targeting effect of the nanomaterials, we used western blotting to examine the expression of stemness markers CD133, CD44, the TGF‐β pathway effector molecules Smad2/3, ferroptosis marker GPX4, and FOXF2. The results indicated that, compared to the control group, Lipo/Erastin/miR/SB significantly inhibited the TGF‐β pathway and stemness in docetaxel‐resistant CRPC cells, while Lipo/Erastin/miR/SB also significantly promoted ferroptosis (Figure 6K).

**FIGURE 7:**
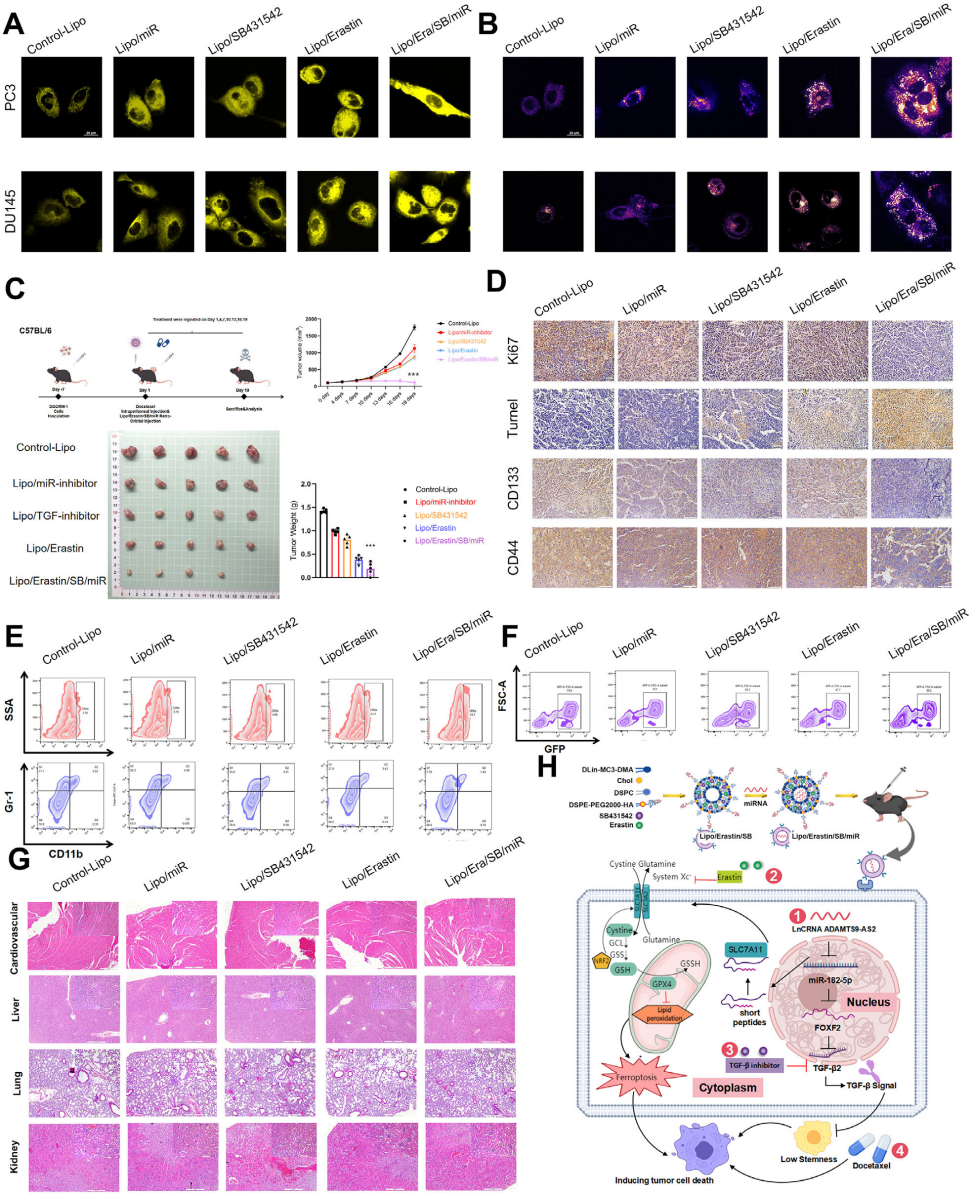
In Vivo Antitumor and Immune Modulatory Effects of Lipo/Erastin/miR/SB Nanoparticles in a DTX CRPC Model. (A) and (B) Fe^2^⁺ and ROS detection assays used to assess the impact of different nanomaterials on ferroptosis induction in CRPC cells. (C) Subcutaneous tumor xenograft experiments conducted to evaluate the impact of different nanomaterials on reversing docetaxel resistance in resistant CRPC cell lines. (D) IHC used to assess the impact of different nanomaterials on tumor cell proliferation, apoptosis, and stemness markers in tumor tissues. (E) Flow cytometry used to evaluate the impact of different nanomaterials on the abundance of CD8⁺ T cells and MDSCs within the tumor. (F) Flow cytometry used to assess the cytotoxic effect of CD8⁺ T cells on tumor cells following co‐culture with nanomaterials. (G) H&E staining used to examine the heart, liver, lung, and kidney tissues of mice to assess the biocompatibility and potential toxicity of the nanomaterials. (H) Schematic illustration of the mechanisms of nanomaterials in modulating tumor progression and overcoming docetaxel resistance. Results were presented as mean ± SD. ns indicated *p* > 0.05; ^*^ indicated *p* < 0.05; ^**^ indicated *p* < 0.01; ^***^ indicated *p* < 0.001.

Subsequently, transmission electron microscopy (TEM), Fe^2+^ ion detection, and ROS detection assays were performed to assess the impact of the nanomaterial Lipo/Erastin/miR/SB on ferroptosis in CRPC cells. The results indicated that, compared to the control group, Lipo/Erastin/miR/SB significantly promoted mitochondrial collapse (Figure S5C), Fe^2+^ accumulation (Figure 7A), increased ROS levels (Figure 7B).

In conclusion, these results suggested that Lipo/Erastin/miR/SB could target and inhibit the stemness of docetaxel‐resistant CRPC cells while promoting ferroptosis, thereby exerting an antitumor effect and delaying docetaxel resistance.

### 3.7 In Vivo Antitumor Effects and Immune Modulation by Lipo/Erastin/miR/SB Nanoparticles in a Docetaxel‐Resistant CRPC Model

To further assess the antitumor efficacy of Lipo/Erastin/miR/SB nanoparticles in vivo, we established a subcutaneous xenograft model using C57BL/6 mice. The treatment regimen and experimental design were illustrated. Compared to the control group, all four treatment groups exhibited varying degrees of tumor volume and weight reduction, with the Lipo/Erastin/miR/SB group demonstrating the most pronounced tumor suppression (Figure 7C). Immunohistochemical (IHC) analysis of tumor tissues revealed strong nuclear Ki67 staining (a marker of proliferation) in the control group, whereas Ki67 expression was markedly reduced in all treatment groups, particularly in the Lipo/Erastin/miR/SB group, indicating suppressed tumor cell proliferative ability in vivo (Figure 7D). Concurrently, the apoptotic marker TUNEL was scarcely detected in the control group but significantly elevated in the Lipo/Erastin/miR/SB group, suggesting enhanced tumor cell apoptosis activity in vivo (Figure 7D). Moreover, expression levels of the cancer stemness markers CD133 and CD44 were substantially downregulated following Lipo/Erastin/miR/SB treatment, indicating effective inhibition of tumor stem‐like properties by the nanoplatform (Figure 7D).

Given that ferroptosis had been reported to elicit localized inflammatory responses and immune cell infiltration—thereby reshaping the tumor immune microenvironment—we further evaluated the immunomodulatory effects of Lipo/Erastin/miR/SB. CRPC was often described as an “immune desert” with high infiltration of myeloid‐derived suppressor cells (MDSCs) and low infiltration of CD8⁺ T cells. Flow cytometry analysis of xenografted tumors showed that treatment with Lipo/Erastin/miR/SB significantly increased the proportion of tumor‐infiltrating CD8⁺ T cells and decreased the abundance of MDSCs (Figure 7E, Figure S5D,E). Furthermore, in vitro cytotoxicity assays confirmed that CD8⁺ T cells isolated from Lipo/Erastin/miR/SB treated mice exhibited enhanced killing capacity against CRPC cells (Figure 7F and Figure S5F). Histological examination of major organs, including the heart, liver, lung, kidney, and spleen, revealed no observable morphological abnormalities or pathological changes, supporting the biosafety of the nanoplatform (Figure 7G and Figure S5G).

Collectively, these findings demonstrated that Lipo/Erastin/miR/SB exerted potent antitumor activity in vivo by suppressing stemness and inducing ferroptosis in docetaxel‐resistant CRPC cells (Figure 7H). Notably, it also remodeled the local immune microenvironment by enhancing immune cell infiltration and cytotoxicity, thus facilitating more effective immune‐mediated tumor clearance. These results supported the potential of Lipo/Erastin/miR/SB as a promising candidate for combination therapy with docetaxel or PD‐1 blockade in patients with advanced, treatment‐resistant CRPC.

## 4 Discussion

Chemotherapy resistance in CRPC remained a significant clinical challenge. Emerging evidence suggested that PCSCs played a pivotal role in mediating chemoresistance. This study elucidated the functional mechanisms by which LncRNA ADAMTS9‐AS2 suppressed cellular stemness and encoded a short peptide that induced ferroptosis, thereby reversing docetaxel resistance in CRPC (Figure 7H).

Our findings demonstrated that PCSCs contributed substantially to docetaxel resistance in CRPC. LncRNA ADAMTS9‐AS2, which was markedly downregulated in PCSCs, was found to inhibit both cellular stemness and docetaxel resistance, with its functionality dependent on the FOXF2/TGF‐β2 axis. Mechanistically, LncRNA ADAMTS9‐AS2 positively regulated the expression of FOXF2 through competitive adsorption of miR‐182‐5p. Subsequently, FOXF2 transcriptionally repressed TGF‐β2, leading to inhibition of the TGF‐β pathway and consequent suppression of PCSC self‐renewal.

Furthermore, we discovered that LncRNA ADAMTS9‐AS2 encoded a functional short peptide that promoted ferroptosis in CRPC cells. This peptide bonded to SLC7A11, impairing its cysteine transport function and consequently depleting intracellular cysteine levels, ultimately inducing ferroptosis and overcoming chemoresistance. Accumulating evidence had revealed a critical role for ferroptosis in modulating docetaxel sensitivity. In prostate cancer, pharmacological induction of ferroptosis markedly enhanced the cytotoxic effects of docetaxel, whereas suppression of ferroptosis diminished its therapeutic efficacy [28]. Recent studies further demonstrated that several non‐coding RNAs contributed to docetaxel resistance by restraining ferroptosis [29, 30]. For example, both the TFAP2C‐driven lncRNA PCAT1 and the m6A‐modified circLPAR3 inhibited ferroptosis in docetaxel‐resistant prostate cancer cells [31], thereby promoting chemoresistance. In contrast to these ferroptosis‐suppressive ncRNAs, our findings identify ADAMTS9‐AS2 as a ferroptosis‐promoting lncRNA.

In terms of clinical translation, lncRNAs already had a successful precedent. Prostate cancer antigen 3 (PCA3/DD3), a highly specific diagnostic lncRNA biomarker, was markedly upregulated in prostate cancer tissues but nearly undetectable in other tissues [32]. It had been developed into a FDA‐approved, non‐invasive urine test (e.g., the Progensa PCA3 assay) to support risk stratification in patients with elevated PSA but negative DRE, guide biopsy decisions, and monitor postoperative recurrence. This clinical application paradigm demonstrated the feasibility of lncRNAs as molecular biomarkers and provided a clear framework for developing novel lncRNA markers. Accordingly, we anticipated that ADAMTS9‐AS2 might likewise serve as a clinically valuable biomarker: it was significantly downregulated in PCSCs and docetaxel‐resistant CRPC and was closely associated with the maintenance of tumor stemness and response to docetaxel. Thus, ADAMTS9‐AS2 might not only aid early detection and prognostic evaluation but also enable prediction of chemotherapy sensitivity and personalized treatment stratification in CRPC. To enhance clinical applicability, future studies should establish an ADAMTS9‐AS2 score and define diagnostic/prognostic thresholds to improve the accuracy of diagnosis and therapeutic response prediction, particularly for CRPC. Moreover, integrating ADAMTS9‐AS2 with liquid biopsy approaches such as exosomal RNA or circulating tumor DNA detection might further optimize its non‐invasive measurement, facilitating its translation from mechanistic discovery to precision clinical management.

To translate these findings into therapeutic potential, we designed and successfully constructed a stem cell–targeting polymeric nanomaterial capable of co‐delivering (1) a TGF‐β inhibitor, (2) a ferroptosis inducer, and (3) a miR‐182‐5p inhibitor. This innovative strategy effectively reversed docetaxel resistance in a CRPC mouse model while exhibiting a favorable biosafety profile. Although numerous studies had investigated nanomaterial‐mediated oxidative stress and ferroptosis for tumor therapy [33, 34, 35], our results provided a particularly promising therapeutic approach for overcoming clinical docetaxel resistance in patients with CRPC.

Immunogenic cell death (ICD) representd a type of regulated cell death characterized by the emission of numerous damage‐associated molecular patterns (DAMPs), which were capable of stimulating antitumor immune responses [36, 37]. Growing evidence indicated that ferroptosis also possessed hallmark features of ICD. During ferroptosis, tumor cells released DAMPs—including calreticulin (CRT), HMGB1, and ATP—that interacted with the receptors CD91, TLR4, and P2RX7 on dendritic cells (DCs), respectively. These interactions facilitated DC activation and subsequently drove the priming and expansion of CD8⁺ T cells [31, 32, 33, 34, 38, 39]. Activated CD8⁺ T cells then secreted IFN‐γ, which enhanced cytotoxic activity and contributed to tumor suppression [40, 41]. In addition, the lipid peroxides generated during ferroptotic processes could augment antigen uptake and presentation by DCs, further strengthening antitumor immunity [42].

Building on the understanding that ferroptosis increased tumor immunogenicity, numerous studies had demonstrated its potential to enhance responses to immunotherapy. For instance, the ferroptosis inducer Cystinase had been shown to synergize with PD‐L1 blockade to amplify T‐cell‐mediated antitumor effects [43]. Similarly, nanoparticles loaded with RSL3 could trigger ICD in melanoma, markedly increasing CD8⁺ T‐cell infiltration and improving the performance of immunotherapy [44]. Consistent with these findings, our in vivo experiments further confirmed the impact of Lipo/Erastin/miR/SB on modulating CD8⁺ T‐cell and MDSC infiltration. These results suggested that ferroptosis induction might help shift prostate cancer from an immunologically “cold” state to a “hot” one, providing a compelling rationale for future combinational immunotherapy approaches.

Tumor organoids [45, 46, 47, 48] had emerged as transformative models in oncology research by faithfully recapitulating the histological complexity, genetic diversity, and tumor microenvironment of primary malignancies. Their unique advantages included enabling personalized drug sensitivity testing (particularly for treatment‐resistant cases), predicting therapeutic responses within clinically actionable time frames (2–4 weeks), and permitting real‐time monitoring of tumor evolution. Compared to traditional models, organoids offered superior biological fidelity while maintaining scalability for high‐throughput screening and genetic manipulation. As patient avatars, they were revolutionizing precision medicine through biomarker discovery, immunotherapy development via immune‐organoid co‐cultures, and direct clinical decision support. To advance precision medicine for prostate cancer patients, our future research would aim to evaluate the therapeutic efficacy and safety of our engineered polymeric material—which delivered TGF‐β inhibitors, ferroptosis inducers, and miR‐182‐5p inhibitors specifically targeting prostate cancer stem cells—using CRPC patient‐derived organoids. This platform would enable systematic assessment of combination therapies incorporating our novel therapeutic with conventional chemotherapeutics, targeted agents, and immunotherapies to identify optimal treatment regimens that maximized clinical benefit for individual patients.

## Conflicts of Interest

The authors declare no conflicts of interest.

## Supporting information




**Supporting File**: advs73442‐sup‐0001‐SuppMat.pdf.

## Data Availability

The data that support the findings of this study are available on request from the corresponding author. The data are not publicly available due to privacy or ethical restrictions.
