# Supplementary material for: ADAMTS9‐AS2 Disrupts Docetaxel‐Resistance in Castration‐Resistant Prostate Cancer via Stemness Suppression and Ferroptosis Induction
Source: Adv Sci (Weinh). 2025 Dec 29;13(10):e20838. doi: 10.1002/advs.202520838 (PMC12915152; doi:10.1002/advs.202520838)
Supplement: Supplementary file 1 — Supporting File: advs73442‐sup‐0001‐SuppMat.pdf. [file ADVS-13-e20838-s001.pdf]

ADAMTS9-AS2 Disrupts Docetaxel-Resistance in Castration-Resistant Prostate Cancer via Stemness Suppression and Ferroptosis Induction

Authors

Ji Liu[1,2†], Yan Gao [3†], Yadong Guo[1,2†], Junfeng Zhang[1,2†], Wentao Zhang[1,2], Zhuoran Gu[1,2] , Haotian Chen[1,2] , Chengqi Jin [1,2] , Peng Luo[4*], Shiyu Mao[1,2*], Yajuan Hao[1,2*], Shuo Shi[1,3*], Xudong Yao[1,2*]

Affiliations

[1]Department of Urology, Shanghai Tenth People's Hospital, School of Medicine, Tongji University, Shanghai, China;
[2]Shanghai Tenth People's Hospital, School of Medicine, Tongji University, Shanghai, China;
[3]School of Chemical Science and Engineering, Department of Laboratory Medicine, Shanghai Tenth People ' s Hospital of Tongji University, Tongji University, Shanghai, 200092, China
[4]The Department of Oncology, Zhujiang Hospital, Southern Medical University, 253 Industrial Avenue, Guangzhou, Guangdong, 510282, People's Republic of China. luopeng@smu.edu.cn

[†]These authors contributed equally to this work.

[*]Address correspondence to: Xudong Yao; yaoxudong1967@163.com and Shuo Shi; shishuo@tongji.edu.cn; Yajuan Hao:haoyajuan1989@126.com; Shiyu Mao:maoshiyu1144@sina.com; Peng Luo:luopeng@smu.edu.cn

Supplementary Materials and Methods

Quantitative Real-Time PCR

Total RNA was extracted using TRIzol reagent (Invitrogen, USA) and reverse transcribed into cDNA using the HiScript III 1st strand cDNA synthesis kit (Vazyme, China). Quantitative real-time PCR (qRT-PCR) was performed using the ChamQ SYBR qPCR Master Mix Kit (Vazyme, China). GAPDH was used as an internal control to normalize relative mRNA expression levels. Results were analyzed using the $2^{-\Delta\Delta Ct}$ method. Primer and plasmid sequences were listed in Table S1 and S2.

Cell Proliferation Assay

The CCK-8 assay was used to evaluate the cytotoxicity of the nanoparticles and their impact on gene expression. Cells were seeded into 96-well plates and incubated overnight to allow for attachment. After treatment with various nanoparticles for a specified period, 10 μL of CCK-8 solution was added to each well. The cells were then incubated at 37°C with 5% CO_2 for 1-4 hours. Absorbance was measured at 450 nm using a microplate reader. Cell viability was determined by comparing the absorbance of treated wells to that of untreated control wells, allowing for an assessment of both cytotoxicity and the potential influence of nanoparticles on gene expression pathways.

Wound Healing Assay

Cells were cultured in 6-well plates, and when confluency reached 100%, a scratch was made, and images were taken at 0 and 24 hours. The migration distance at 24 hours was calculated by subtracting the distance between the scratch edge at 0-hour and the migration front at 24-hour using ImageJ software. All experiments were performed in triplicate.

Migration and Invasion Assay

This experiment was performed in a transwell plate with an 8.0 μm polycarbonate membrane (Corning, USA). Cells were mixed with serum-free medium and placed in the upper chamber. A total of 500 μL of complete medium was added to the lower chamber. The cells were incubated at 37°C with 5% CO_2. After 48 hours, the cells were stained with crystal violet, and images were captured. Each experiment was performed in triplicate.

Luciferase Reporter Assay

The association between lncRNA and miRNA was predicted using various databases, including RAID (http://www.rna-society.org/raid/index.html), miRDB (http://www.mirdb.org/), mirDIP (http://ophid.utoronto.ca/mirDIP/), TargetScan (http://www.targetscan.org), and miRTarBase (http://mirtarbase.mbc.nctu.edu), combined with co-expression analysis data from the TCGA-PRAD dataset. The sequences of the wild-type and mutated types of ADAMTS9-AS2 were synthesized by Aibosi (Shanghai, China). Luciferase activity was measured using a dual-luciferase reporter gene kit (Yeasen, Shanghai, China). Subcellular localization of lncRNAs was analyzed using the lncATLAS database (http://lncatlas.crg.eu/) to predict potential regulatory mechanisms of lncRNAs. Comparative analysis of the predicted lncRNAs with differentially expressed miRNAs in the TCGA database was used to identify candidate target genes.

Fluorescence in Situ Hybridization (FISH)

The subcellular localization of ADAMTS9-AS2 was detected using a FISH kit (BIS-P0001, Guangzhou Boxin Biotechnology Co., Ltd., Guangzhou, China) following the manufacturer's instructions. The experiment was performed in triplicate using a Zeiss LSM880NLO confocal microscope (2 + 1 with BIG, Leica Microsystems, Wetzlar, Germany).

Western Blot

Total protein was extracted using SDS lysis buffer (Beyotime, Nantong, China). Protein concentrations were measured using the BCA Protein Assay Kit (Beyotime, Nantong, China). A total of 40 μL of protein was loaded into a 12% SDS-PAGE gel. Polyvinylidene fluoride (PVDF) membranes (Millipore, Burlington, MA, USA) were used for protein transfer. Non-specific binding was blocked with 5% milk. Primary antibodies were incubated overnight at 4°C. The following day, membranes were washed three times for 10 minutes each time with PBST. Secondary antibodies were then applied and incubated for 1 hour at room temperature. The membranes were

washed three times with PBST for 10 minutes each time. Protein bands were detected using a silver stain detection system (Beyotime, Nantong, China) and ECL (Yeasen, Shanghai, China). The details of the antibodies used in this study were shown in Table S3.

Immunohistochemistry (IHC) Analysis

Tissues were fixed and embedded using 4% paraformaldehyde (PFA). After deparaffinization, dehydration, antigen retrieval, and blocking, tissue sections were incubated with primary antibodies overnight at 4°C. Following primary antibody incubation, sections were treated with biotinylated goat anti-rabbit IgG for 20 minutes at room temperature, followed by incubation with streptavidin-horseradish peroxidase for 30 minutes. Finally, the tissues were stained with diaminobenzidine-H_2O_2 and hematoxylin.

Enrichment Analysis

Biological pathways between clusters were examined using gene set variation analysis (GSVA). Gene Ontology (GO) was used to describe the biological processes (BP), molecular functions (MF), and cellular components (CC) of genes. Pathway annotation was carried out using the Kyoto Encyclopedia of Genes and Genomes (KEGG). Additionally, the FDR threshold (0.05) was applied to the c5.cp.kegg.v5.2.symbols.gmt reference gene set.

Morphological characterization of liposomes:

The particle size and potential of LPs were measured by a potentiometer , and transmission electron microscopy (TEM) were used to determine the morphology of the LPs.

Stability of Liposomes:

The prepared liposomes were stored in a refrigerator at 4 °C, and the particle size and zeta potential of the liposomes were measured daily for a period of 7 days.

Supplementary Figure

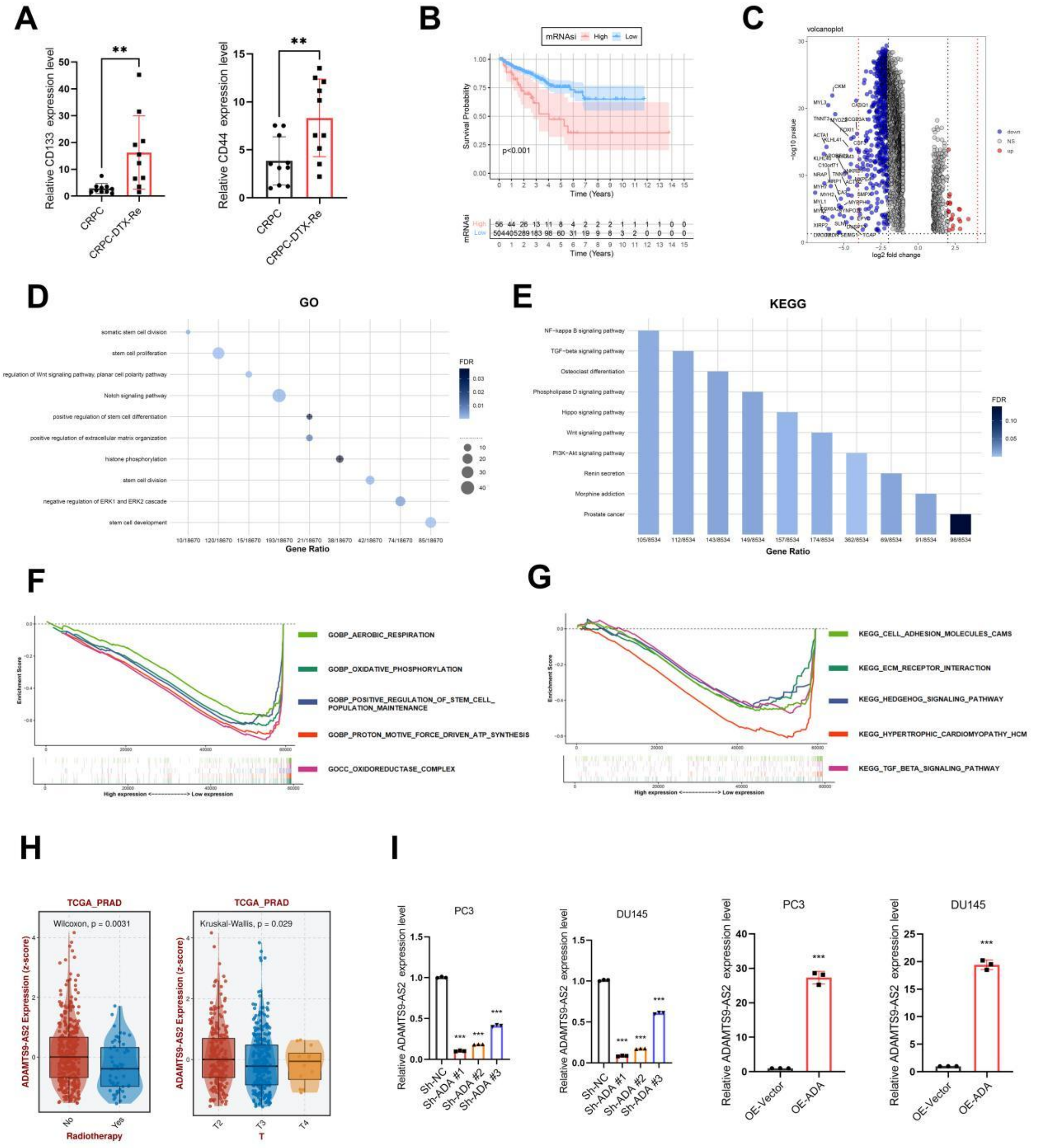

Figure S1 Docetaxel Resistant CRPC Exhibiting a Positive Correlation with Tumor Cell Stemness. (A) RT-qPCR validation of stemness marker levels in 10 docetaxel sensitive and resistant CRPC samples separately. **(B)** KM survival curves exploring the correlation between mRNAsi and prognosis in PCa patients. **(C)** Volcano plot depicting differentially expressed genes between the high and low mRNAsi groups in the TCGA-PRAD dataset. **(D) and (E)** GO and KEGG pathway enrichment analyses of mRNAsi-related differentially expressed genes. **(F) and (G)** GSEA results for ADAMTS9-AS2, showing enrichment in GO and KEGG pathways. **(H)** Correlation analysis between ADAMTS9-AS2 expression, radiotherapy efficacy, and T-stage in PCa patients. **(I)** RT-qPCR analysis showing the efficiency of ADAMTS9-AS2 knockdown and overexpression efficiency in PC3 and DU145 cell lines. Results were presented as mean ± SD. ns indicated P > 0.05; * indicated P < 0.05; ** indicated P < 0.01; *** indicated P < 0.001.

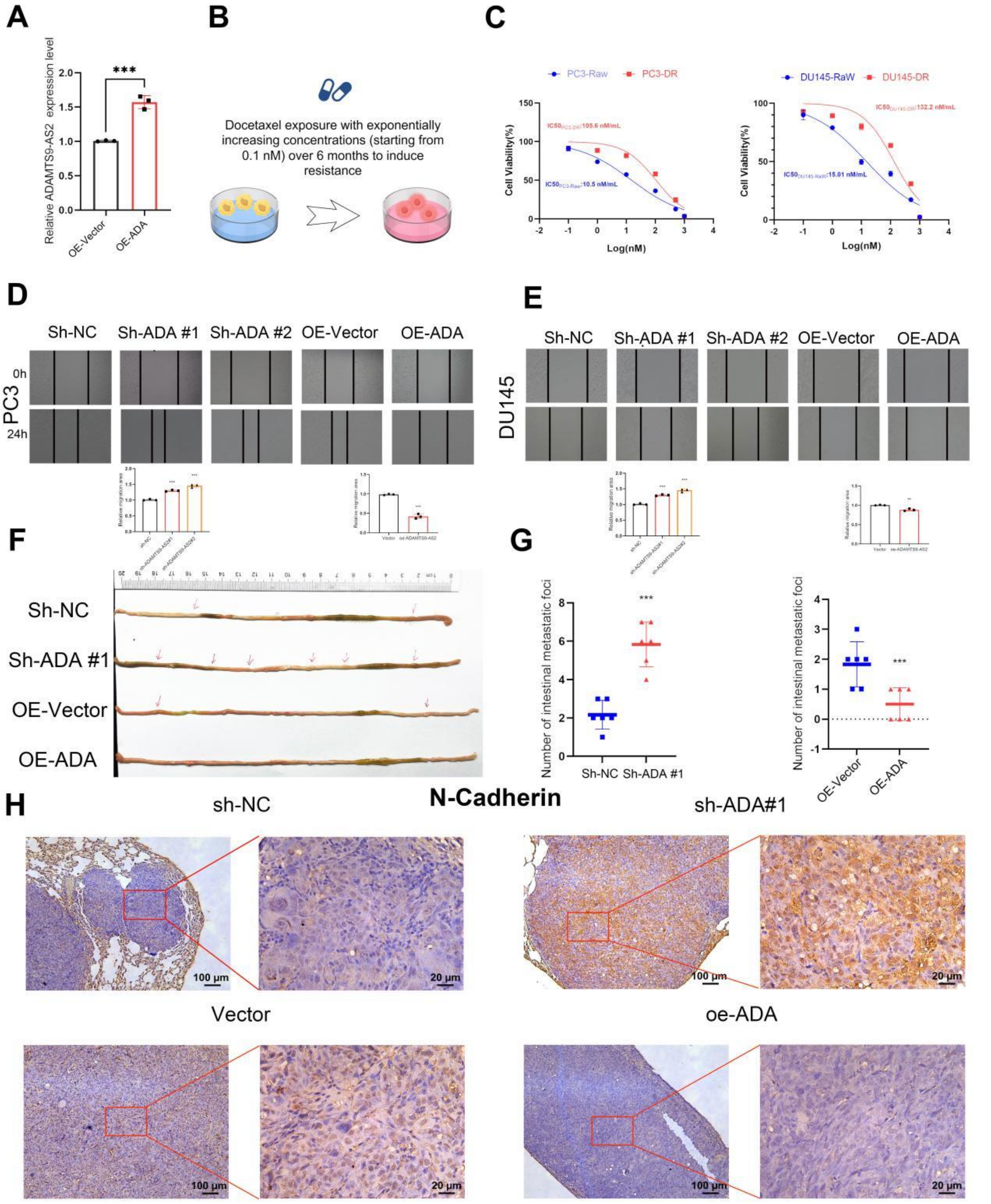

Figure S2 Impact of ADAMTS9-AS2 on Docetaxel Resistance and Malignant Progression in CRPC Cells. (A) RT-qPCR validation of ADAMTS9-AS2 overexpression efficiency in mouse-derived cell lines. **(B)** Schematic diagram illustrating the construction process of the docetaxel resistant cell line. **(C)** CCK-8 assay determining the IC50 values of docetaxel resistant CRPC cell lines. **(D) and (E)** Wound healing assays evaluating changes in the migration ability of CRPC docetaxel resistant cells following ADAMTS9-AS2 knockdown or overexpression. **(F) and (G)** In vivo validation of ADAMTS9-AS2 modulation on CRPC lung metastasis. **(H)** IHC analysis showing the impact of ADAMTS9-AS2 modulation on the expression of EMT marker N-Cadherin in vivo. Results were expressed as mean ± SD. ns indicated $P > 0.05$; * indicated P <

0.05; ** indicated P < 0.01; *** indicated P < 0.001.

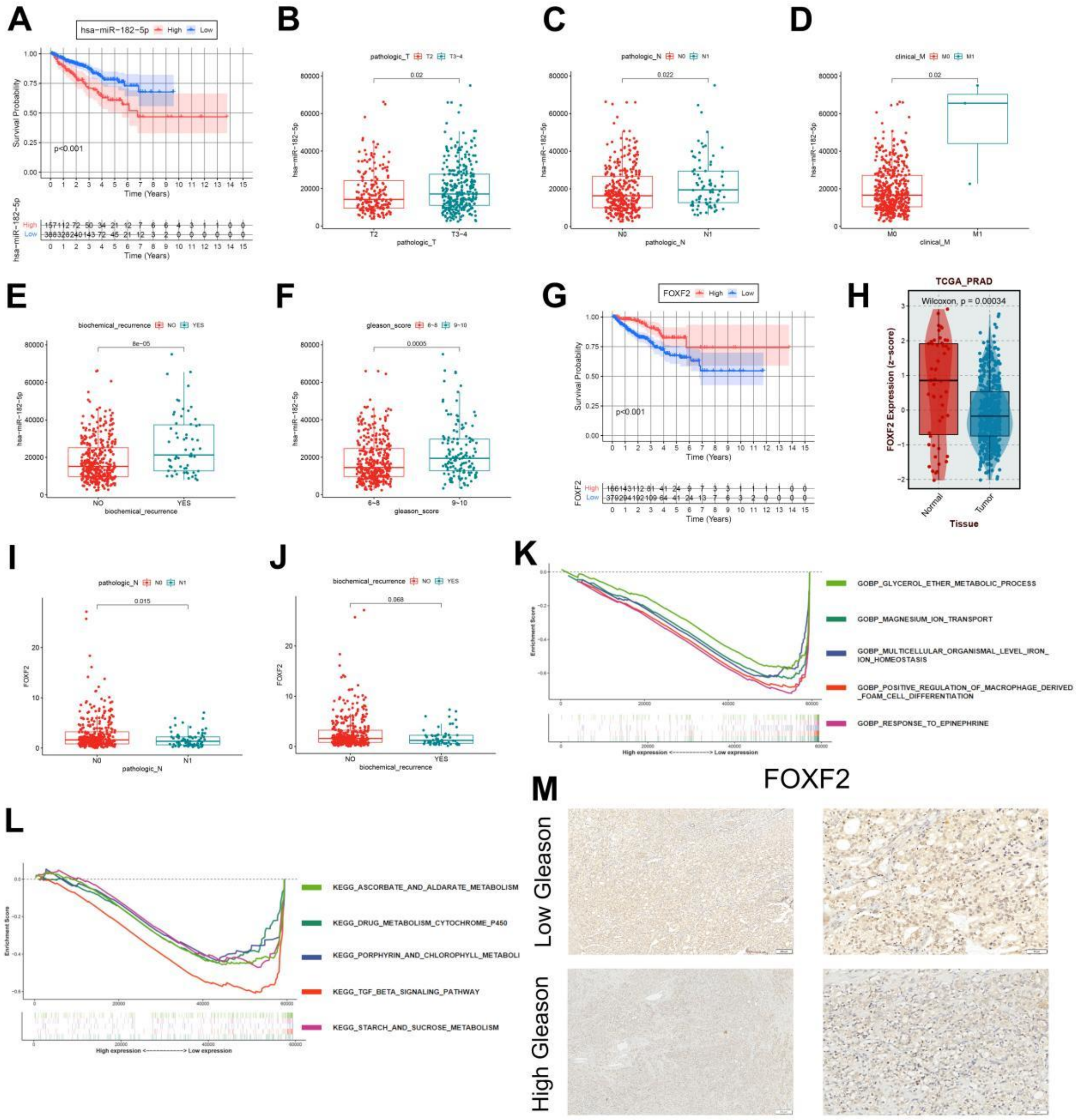

Figure S3 The Clinical Features of miR-182-5p and FOXF2 in TCGA-PRAD Cohort. (A) KM survival curve assessing the correlation between miR-182-5p expression and prognosis in PCa patients. **(B)-(F)** Box plots investigating the association between miR-182-5p expression and clinical features, including T stage, N stage, M stage, biochemical recurrence, and Gleason score in TCGA-PRAD cohort. **(G)** KM survival curve evaluating the prognostic value of FOXF2 expression in PCa patients. **(H)** Box plot validating the expression level of FOXF2 in normal versus tumor tissues in TCGA-PRAD cohort. **(I) and (J)** Box plots exploring the correlation between FOXF2 expression and clinical features, including N stage and biochemical recurrence in TCGA-PRAD cohort. **(K) and (L)** GSEA results for ADAMTS9-AS2 showing the enriched GO and KEGG pathways. **(M)** Expression levels of FOXF2 in normal versus PCa tumor samples. Results were presented as mean ± SD. ns indicated P > 0.05; * indicated P < 0.05; ** indicated P < 0.01; *** indicated P < 0.001.

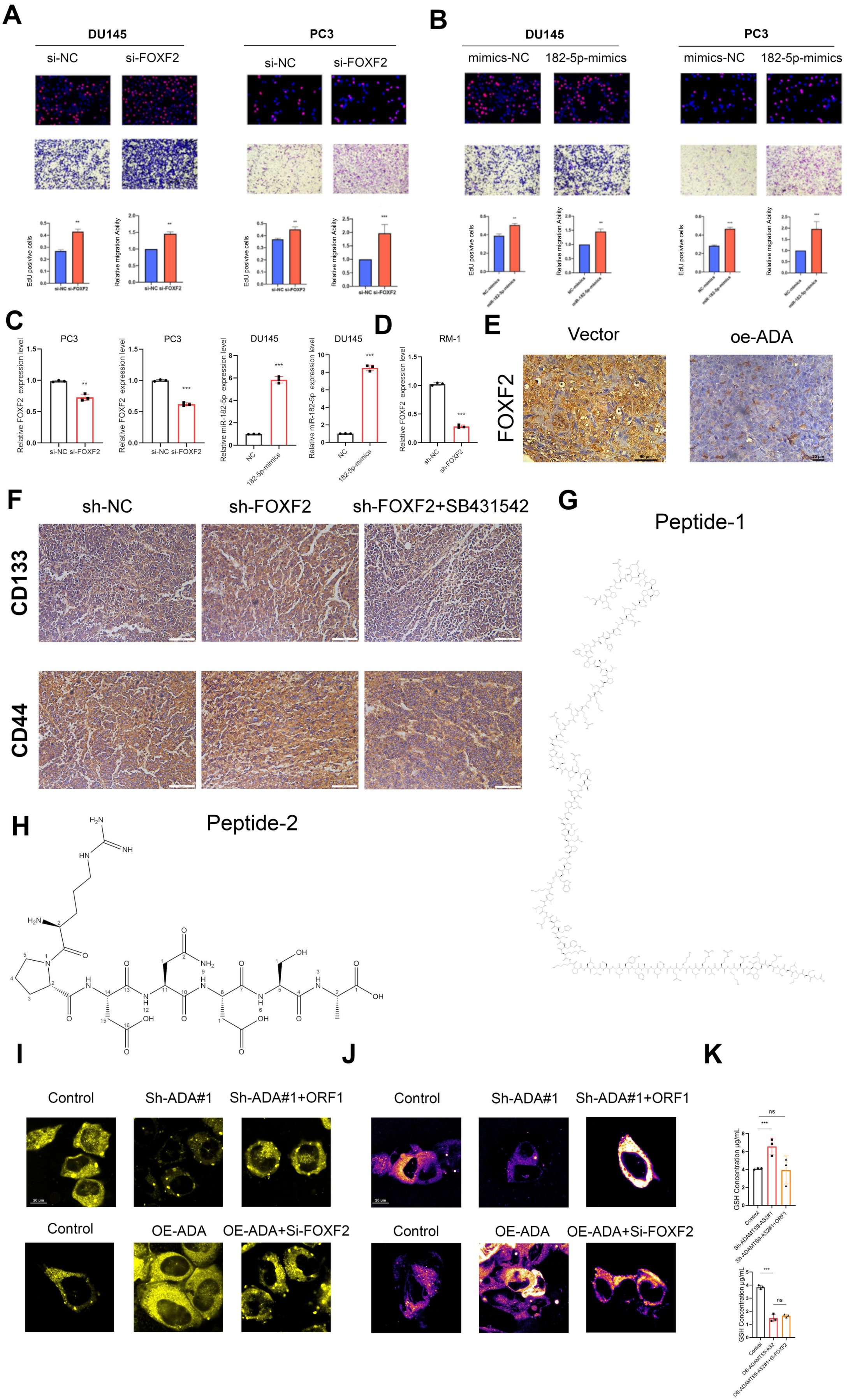
A
DU145
si-NC
si-FOXF2
PC3
si-NC
si-FOXF2
B
DU145
mimics-NC
182-5p-mimics
PC3
mimics-NC
182-5p-mimics
C
PC3
PC3
DU145
DU145
si-NC si-FOXF2
NC
182-5p-mimics
D
RM-1
sh-NC
sh-FOXF2
E
Vector
oe-ADA
FOXF2
F
sh-NC
sh-FOXF2
sh-FOXF2+SB431542
CD133
CD44
G
Peptide-1
H
Peptide-2
I
Control
Sh-ADA#1
Sh-ADA#1+ORF1
Control
OE-ADA
OE-ADA+Si-FOXF2
J
Control
Sh-ADA#1
Sh-ADA#1+ORF1
Control
OE-ADA
OE-ADA+Si-FOXF2
K
GSH Concentration μg/mL
ns
***

Figure S4 Impact of FOXF2 and miR-182-5p on CRPC Progression and Stemness. (A) EdU and Transwell assays assessing the effects of FOXF2 knockdown or miR-182-5p overexpression on the proliferation and invasion ability of CRPC cell lines. **(C) and (D)** Verification of the efficiency of FOXF2 knockdown and/or miR-182-5p overexpression by RT-qPCR. **(E)** IHC analysis validating the effect of ADAMTS9-AS2 overexpression on FOXF2 expression in vivo. **(F)** IHC analysis showing the expression of stemness markers after SB431542 rescue upon FOXF2 knockdown in CRPC cells. **(G) and (H)** Two-dimensional structural diagrams of short peptides encoded by ADAMTS9-AS2 ORF1 and ORF2.**(I-K)** Rescue experiments investigate how modulation of the ADAMTS9-AS2/FOXF2 axis and the ADAMTS9-AS2/ORF1 axis affects ferroptosis in CRPC cells. Results were presented as mean ± SD. ns indicated $P > 0.05$; * indicated $P < 0.05$; ** indicated $P < 0.01$; *** indicated $P < 0.001$.

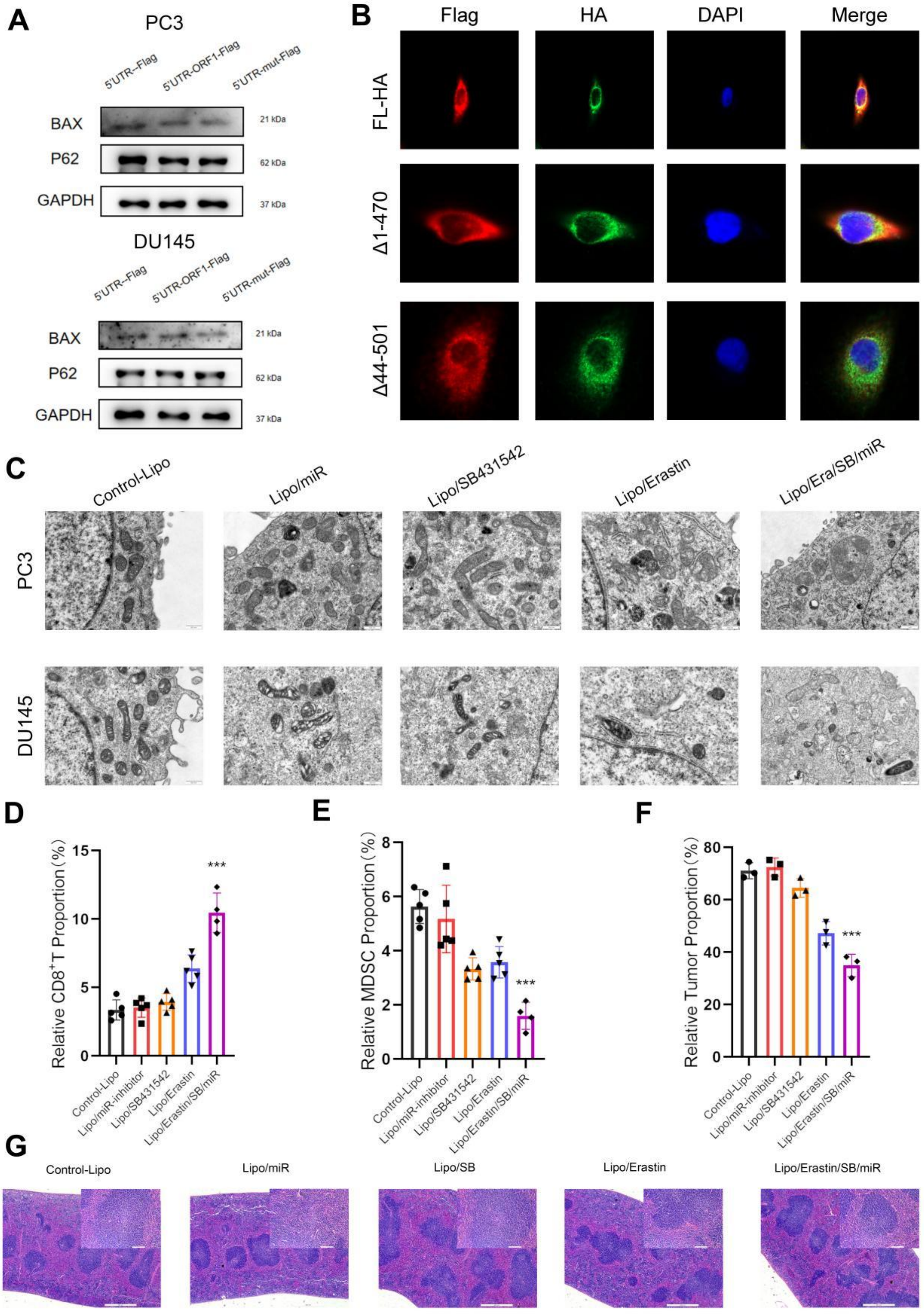

Figure S5 Impact of ADAMTS9-AS2 ORF1 and Targeted Nanomaterials on CRPC. (A) WB analysis of apoptosis and autophagy marker expression following overexpression of ADAMTS9-AS2 ORF1 in CRPC cell lines. **(B)** Immunofluorescence staining demonstrating the colocalization states of Short Peptide 1 with truncated form of SLC7A11 in CRPC cells. **(C)** TEM

images showing the impact of various nano-materials on mitochondrial morphology in CRPC cells. **(D) and (E)** Bar graphs depicting the correlation between the infiltration of $CD8^{+}$ T cells and MDSCs in vivo following treatment with different nano-materials. **(F)** Bar graph illustrating the effects of different nano-material treatments on the cytotoxic activity of $CD8^{+}$ T cells against CRPC cells in vitro. **(G)** H&E staining showing the impact of nano-material treatments on mouse spleen tissue.

Supplementary Table

Name	Sequence
sh-ADAMTS9-AS2#1	5’-GCATGACGCAACTTTGCTATA-3’
sh-ADAMTS9-AS2#2	5’-GCATGGGATGTATCTCCATTA-3’
sh-ADAMTS9-AS2#3	5’-GGTAGGAACACGATCCTAAAG-3’
oe-circCNOT6L	5’-AAACTTGACGTACACACGCAGTCCTATCCCTACGGTCCT<br>GGAATTGGGGGTTACTATCTTGGAATCTAGGGGCACTCCA<br>GGCTCTGGGCTCAGACGGCTGGCTTCTGCCTACCCGAGCCT<br>TAACCTTTCAAGGACCAGAAGGATTCCAGAGCTCTTGCCCT<br>AGGTCCTGGGGCAGCGATGACTCACTGCAGCACCCCCTCC<br>CACTTCGCCAAGCTGCCGTCTCCGCCCACCCCCAAACAATC<br>TCGACAGCGCATTTCGGGAGCCACGGCTCCGGGCGCTTTGC<br>TGGGGGCTAAAGGGGTTTATCCCTTTCCTTGAATCCCAGCA<br>GGCTAGAACTACCCCCTCCCAGTCTTCAGGCTTGCCACGCT<br>CTCCACCCGATCCTTCCATTGAAAGGCAGAGAAGGAAGGA<br>TGTGCTTGGGAACTTTAAGACCCACGAACGACAGCGCACT<br>GATGGAGCAGCCCAGTGTCTGGGGCAAAGTCCTCGAGGTT<br>CATTCATTCAGGAAGCCTCTGACCAGCCTTTACCATGCGCT<br>GAGTGAGAAGCTGAGGATAAGGAAAGAGCAAGACCCCCA<br>AGAAACCCTGATGTCTGGCTGAAAGCCGAAGCATGACGCA<br>ACTTTGCTATATTTCTCTCCAACAAGGATTTGTATATTTTCG<br>CTTTCTCCTCAAGTAACACCTGGACCTGCTCCTTTCCCTTCA<br>AACGCTGAGGGCTCAGTCTCCAAGTTCCTTTATGAAACAGG<br>GTGTACCATCAGAGACGCAGGTATTTATTGAAACCTGCTTT<br>GTGCTGGGCAATGTGCCAGATGTTGGGGATAAAATGGAGA<br>CTTCAGAAATGAAGATGCAAAGACAACAGGGGAAACCATA<br>TATGTTTATGGACAGTCATGCCAAAGTATGATTGGAAGAC<br>AAAAAGATTTTTGGCCAATTCACTGCTCACTAACATCTCCA<br>TCAAAGGGAAAAACACACATTGTAAAATAAGAATTCTTCA<br>AAGACAACTTGAAGACATCTGGGAATTCTGAAGCCTGAAA<br>ATTCCATGAGTTCATAGCAGTGTTCACTAATTCCACTGATC<br>TAAAGACAAAAAGGAGAGTCAACTGCGTGCCCAAACTCAT<br>TTCTGATGATTTCTTCCATCCTAACTTTTCTGCCTCTCTGGG<br>GGTGGTTATGCCTGTGAAAAACATTGAAGCTGTCAGGAAC

	AGAGTCGGCTTTCAAGATTGGAATGTCAAAGTAGAGTCTTC<br>TTTTCCTCAGACCAGAAGGGGCTTGGTTGGGTAAGAGTTTC<br>CAGATCTAAATGGAGACAGGACTGGGATATAGGAGCTTGT<br>ACGGATAAACAACACAAACACCTTGGGTAAGTGATACACC<br>AAGAGAGATCAAAATAGGGTAGGAACACGATCCTAAAGCT<br>ATGTGAGATACGAAGCCAAGAGACCCTGTCTACAGGCTGA<br>TATGTATTGCAACCAGAGGCCACCTACACTCAAGAAAATT<br>CTAAATGATGTAATAGGTTGTGGACACTTTCTTGTAAAAAT<br>GATCGATATTTTTTTCTTATAGGGATAAAGCTTAAAAGCAT<br>GGGATGTATCTCCATTAGGAAATATGAAGTGAGTTGGTAT<br>ATTACAGGTAGGTTAGACTGAGATAATTATAAAACTGGGC<br>TGATTTTACATAGAGAAGAACTTGATTCCTAAAGGTGTTGA<br>AGGACAAGATGGGGACAGTTATTGAAGTGTTCAGGTGCTG<br>TCCTTTGTAGTCTGTAGCCTATTAAAATAATACACAACTGA<br>ACACAGACCCAAGGAAAGGGGTTCTACTCATCGCTAGAAT<br>GTAAACTCTAAGAAGATAAGGATTTTGTCTGCTTCATTTAT<br>TACTGTATTCCCAGCTCCTAAAAGAGTGTCCAGCACTTAGT<br>ATCTGTTGGTAAGTACCTGTTGAATTAATTAACCAGTGATG<br>AGACTTTGGAAAGCAGCTAGTCCATCTGTAAGATTGTTTTC<br>TCATCTGTGAGATGAGTCAGTTAGATTAGATGTTTTCTAAG<br>CTTATTCTAGTTTTAAAATTGTATATTTCTATAAGAAATGAT<br>AAATGAAAAATGTCCATTCCTTTTTCATCTCCATCACCCAT<br>CTCACATCTTCCTTCAGTGCCTATGTGATCTGTTTGAGTATA<br>ACCAGGTGTACAAAAGTACTAAGTGATATGAGATGACTTC<br>AGTAGTCCAGGGACTCTTTCTTCCCCCACTCTGTAAAGAGT<br>TATTTGCTTAATTTAATGTACATTATAAAAACAGA -3’
Sh-FOXF2	5’GCGTCATGTGAACGGAAAG3’

Table S1:The sequense of siRNA , shRNA and oe-RNA

**Name**	**Sequence**
ADAMTS9-AS2	Forward: 5’-TTTACCATGCGCTGAGTGAG-3’<br>Reverse: 5’-AAAGTTGCGTCATGCTTCGG-3’
GAPDH	Forward:5’-GGGAAATTCAACGGCACAGT-3’<br>Reverse: 5’AGATGGTGATGGGCTTCCC-3’
SLC7A11	Forward: 5’- TCTCCAAAGGAGGTTACCTGC-3’<br>Reverse: 5’ AGACTCCCCTCAGTAAAGTGAC-3’
FOXF2	Forward: 5’-TGCACTCCAGCATGTCCTCCTA-3’<br>Reverse: 5’-CGCTAGCTGAGGGATGGAAAGA-3’
miR-182-5p	Forward:5’-ACACTCCAGCTGGGUCACACUCAAGAUGGUAA-3’<br>Reverse:5’-CTCAACTGGTGTCGTGGAGTCGGCAATTCAGTTGA GTTGCC AAA-3’
CD133	Forward: 5’-CACTACCAAGGACAAGGCGTTC-3’<br>Reverse: 5’-CAACGCCTCTTTGGTCTCCTTG-3’

CD44	Forward: 5'-CCAGAAGGAACAGTGGTTTGGC-3'<br>Reverse: 5'-ACTGTCCTCTGGGCTTGGTGTT-3'
Nanog	Forward: 5'-CTCCAACATCCTGAACCTCAGC-3'<br>Reverse: 5'-CGTCACACCATTGCTATTCTTCG-3'
OCT4	Forward: 5'-CCTGAAGCAGAAGAGGATCACC-3'<br>Reverse: 5'-AAAGCGGCAGATGGTCGTTTGG-3'
KLF4	Forward: 5'-CATCTCAAGGCACACCTGCGAA-3'<br>Reverse: 5'-TCGGTCGCATTTTTGGCACTGG-3'

Table S2:The list of Primer

**Name**	**Brand**	**Number**	**Working concentration**
GAPDH	Abcam	ab8245	1/2000
PCNA	CST	#13110	1/1000
Snail	Abcam	ab216347	1/1000
Cadherin	Abcam	ab76011	1/5000
Vimentin	Abcam	ab92547	1/1000
E-Cadherin	Abcam	ab40772	1/1000
FOXF2	HUABIO	RT1230	1/1000
SLC7A11	Abcam	ab307601	1/1000
GPX4	Abclonal	A11243	1/1000
TGF-β1	Abclonal	A2124	1/2000
TGF-β2	Abclonal	A25496	1/1000
TGF-β3	Abclonal	A8460	1/1000
Na+/K+ ATPase	HUABIO	RT1412	1/1000
BAX	Abcam	ab32503	1/1000
P62	HUABIO	HA721171	1/1000

Table S3:The detail of antibody in this experiment
